# Supplementary figures and images for: Evolutionary and functional analyses reveal a role for the RHIM in tuning RIPK3 activity across vertebrates
Source: eLife. 2025 May 28;13:RP102301. doi: 10.7554/eLife.102301 (PMC12119088; doi:10.7554/eLife.102301)

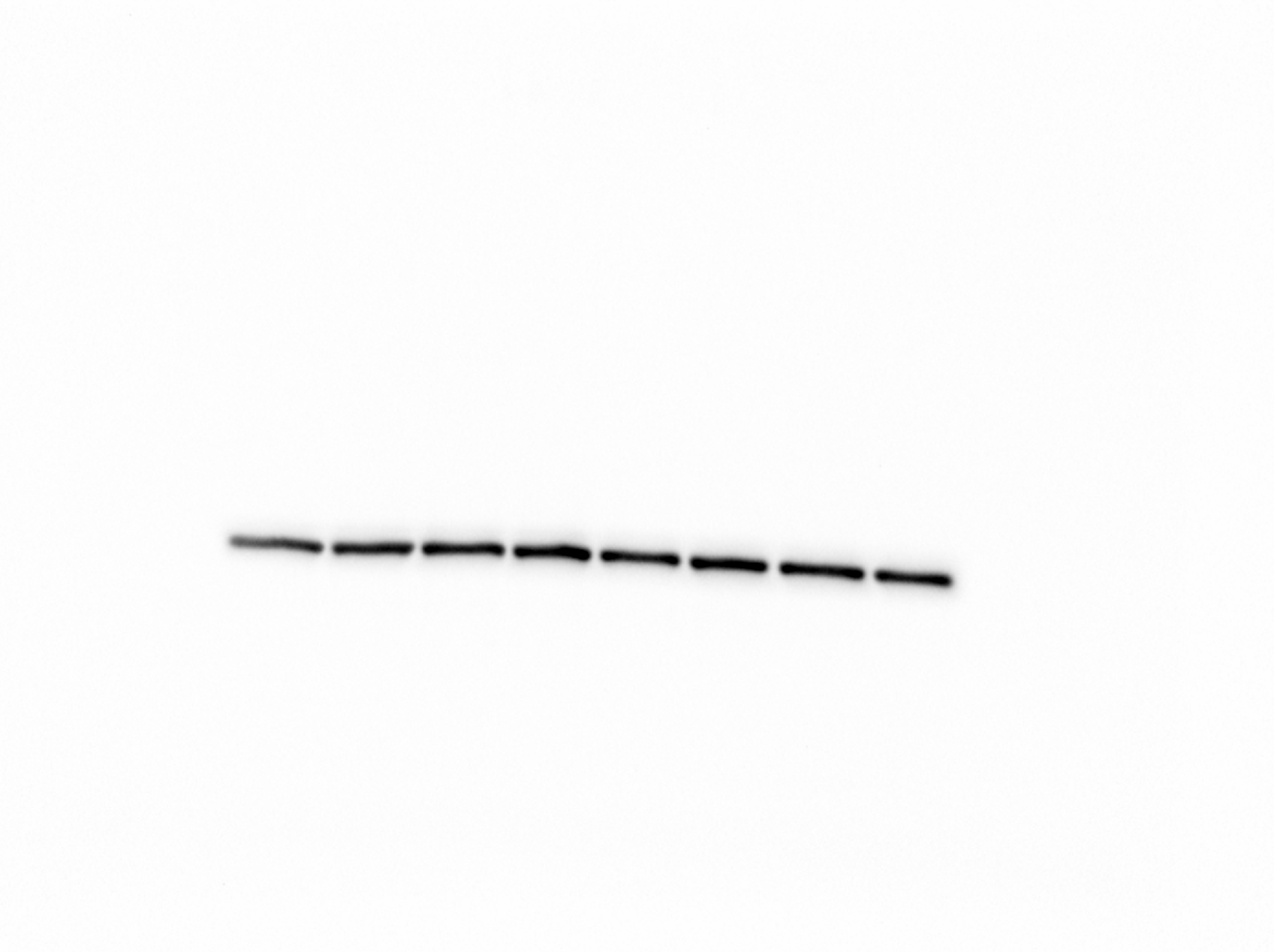

Supplement: Figure 1—figure supplement 2—source data 1. [file elife-102301-fig1-figsupp2-data1.zip › Figure 1-figure supplement 2A-GAPDH.jpg]

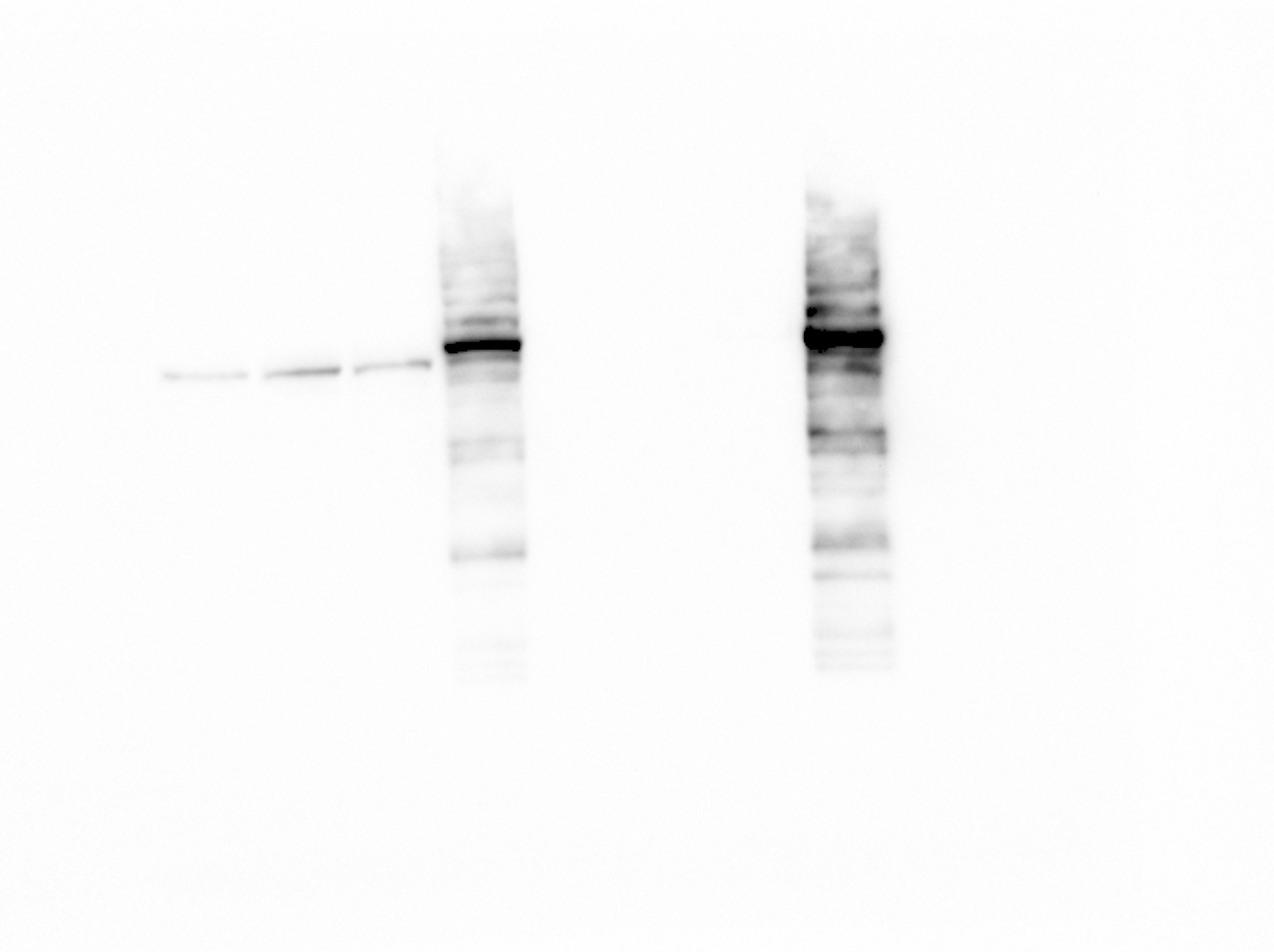

Supplement: Figure 1—figure supplement 2—source data 1. [file elife-102301-fig1-figsupp2-data1.zip › Figure 1-figure supplement 2A-RIPK1.jpg]

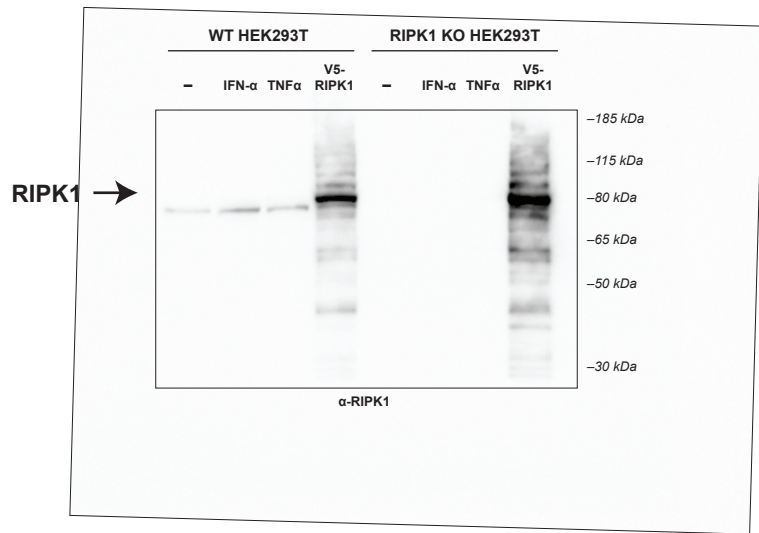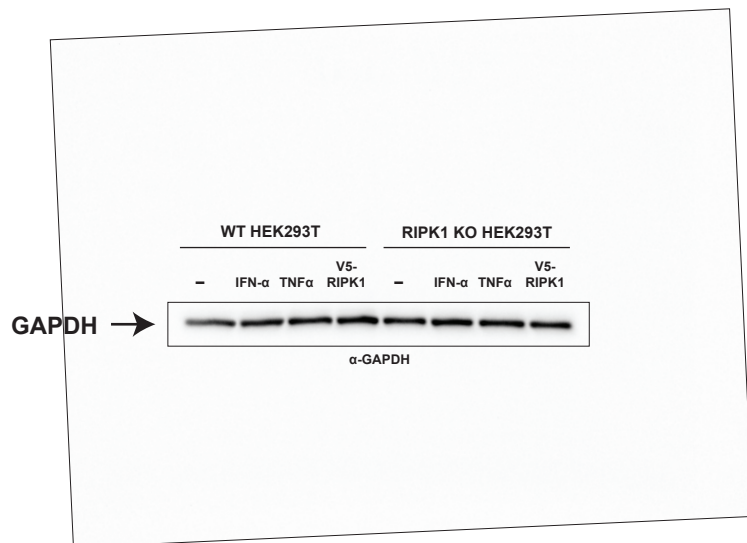

Supplement: Figure 1—figure supplement 2—source data 2. [file elife-102301-fig1-figsupp2-data2.zip › Figure 1-figure supplement 2-source-data2.pdf]

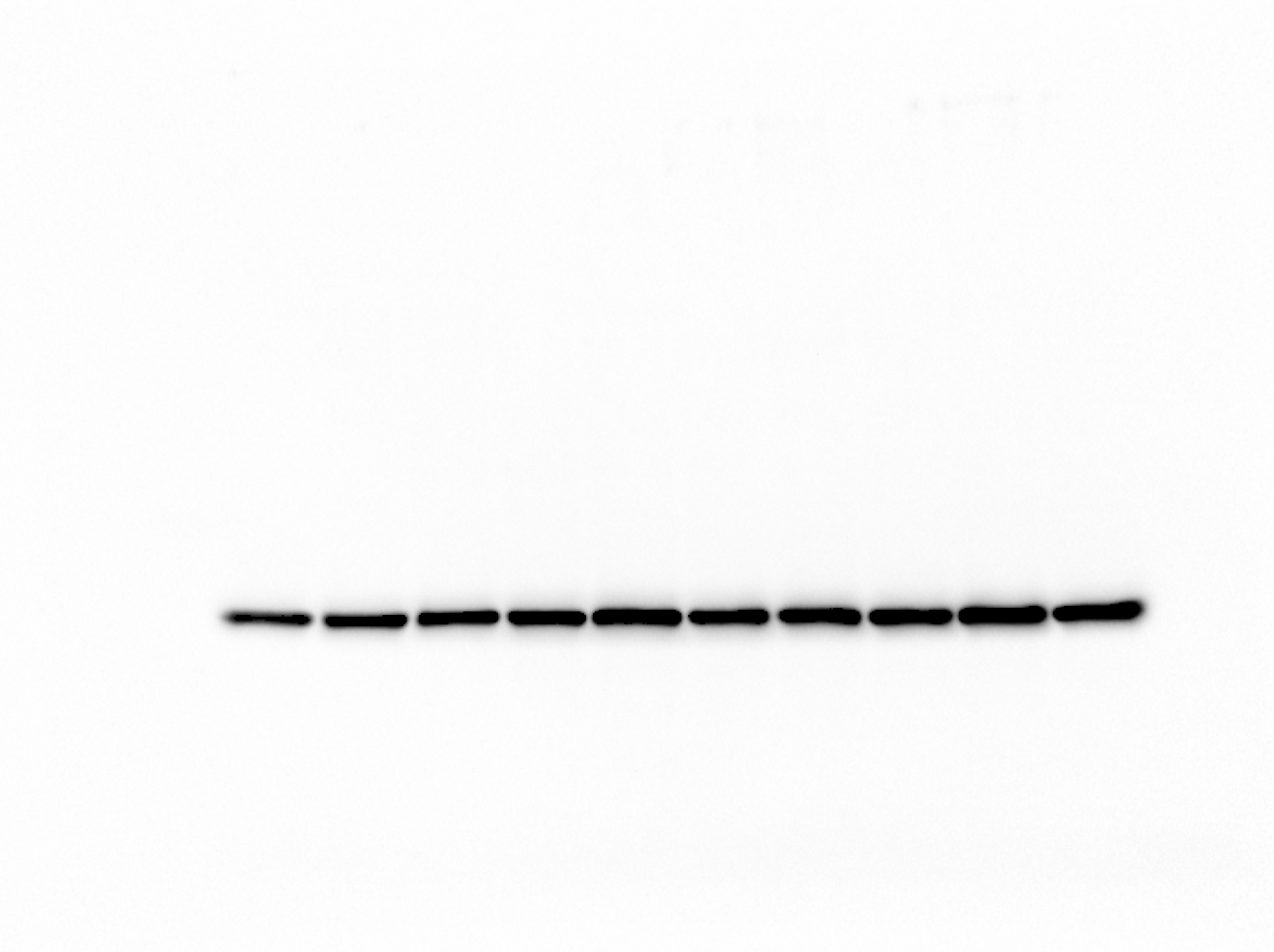

Supplement: Figure 1—figure supplement 3—source data 1. [file elife-102301-fig1-figsupp3-data1.zip › Figure 1-figure supplement 3A-GAPDH.jpg]

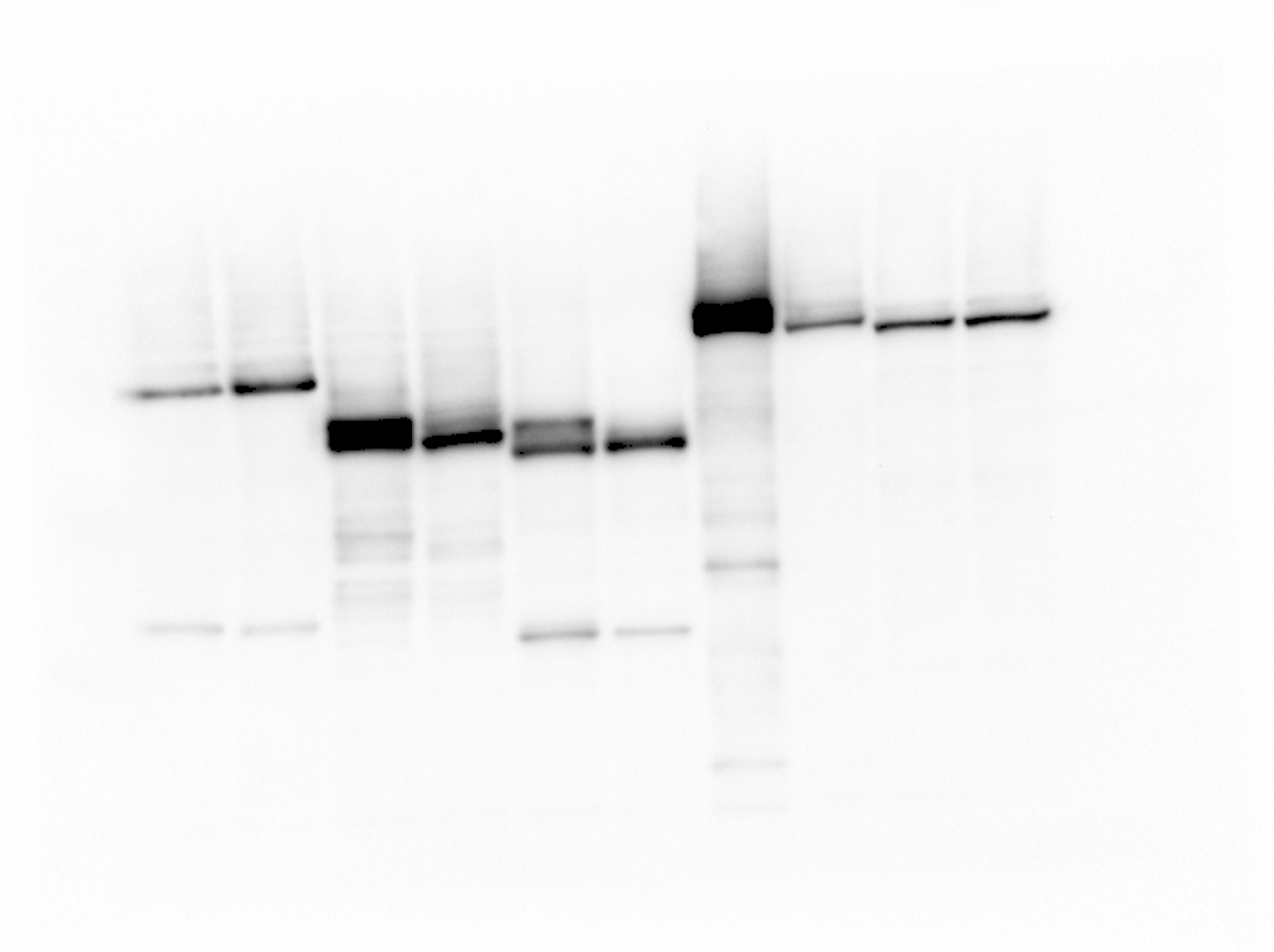

Supplement: Figure 1—figure supplement 3—source data 1. [file elife-102301-fig1-figsupp3-data1.zip › Figure 1-figure supplement 3A-V5.jpg]

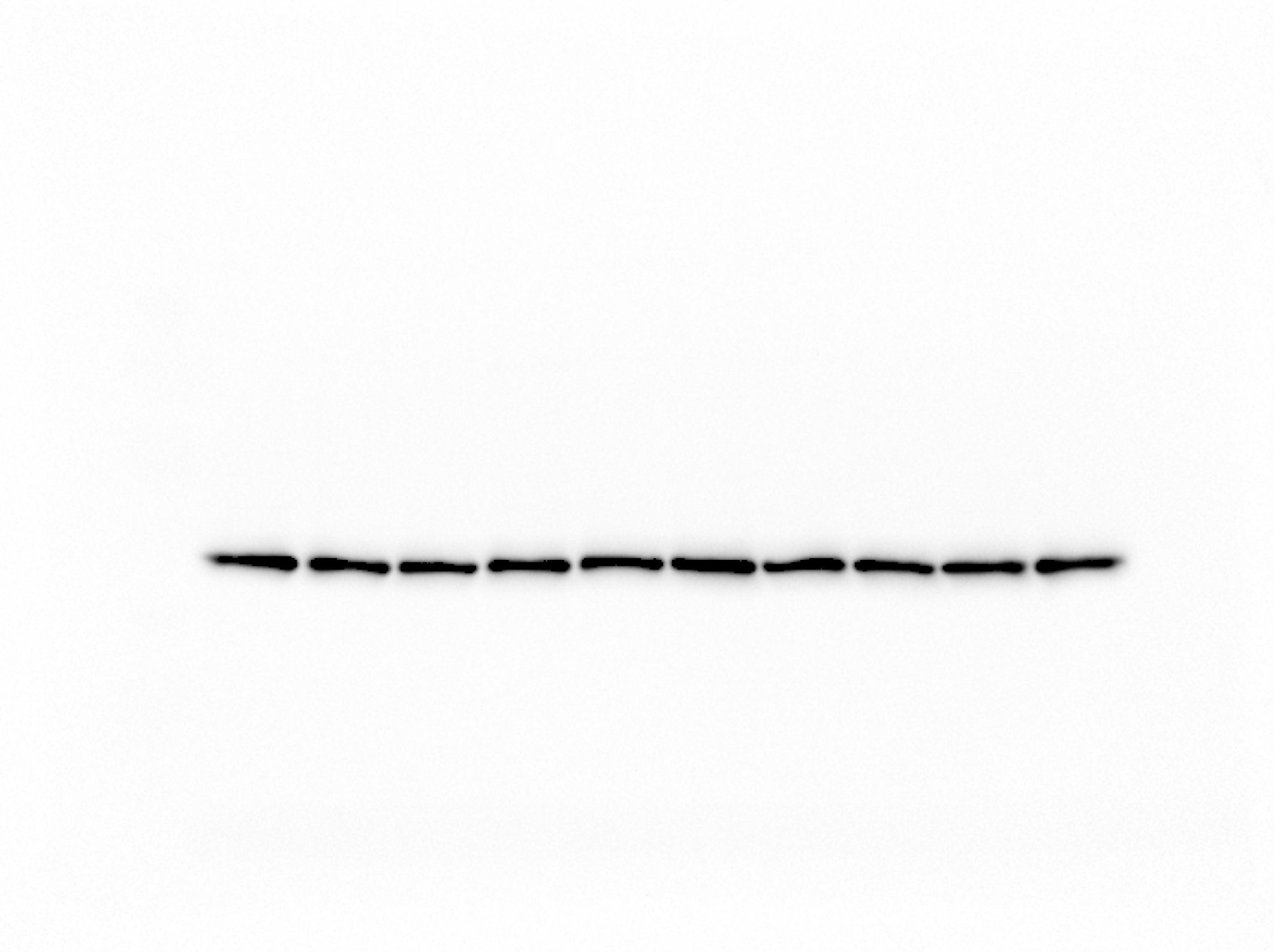

Supplement: Figure 1—figure supplement 3—source data 1. [file elife-102301-fig1-figsupp3-data1.zip › Figure 1-figure supplement 3B-GAPDH.jpg]

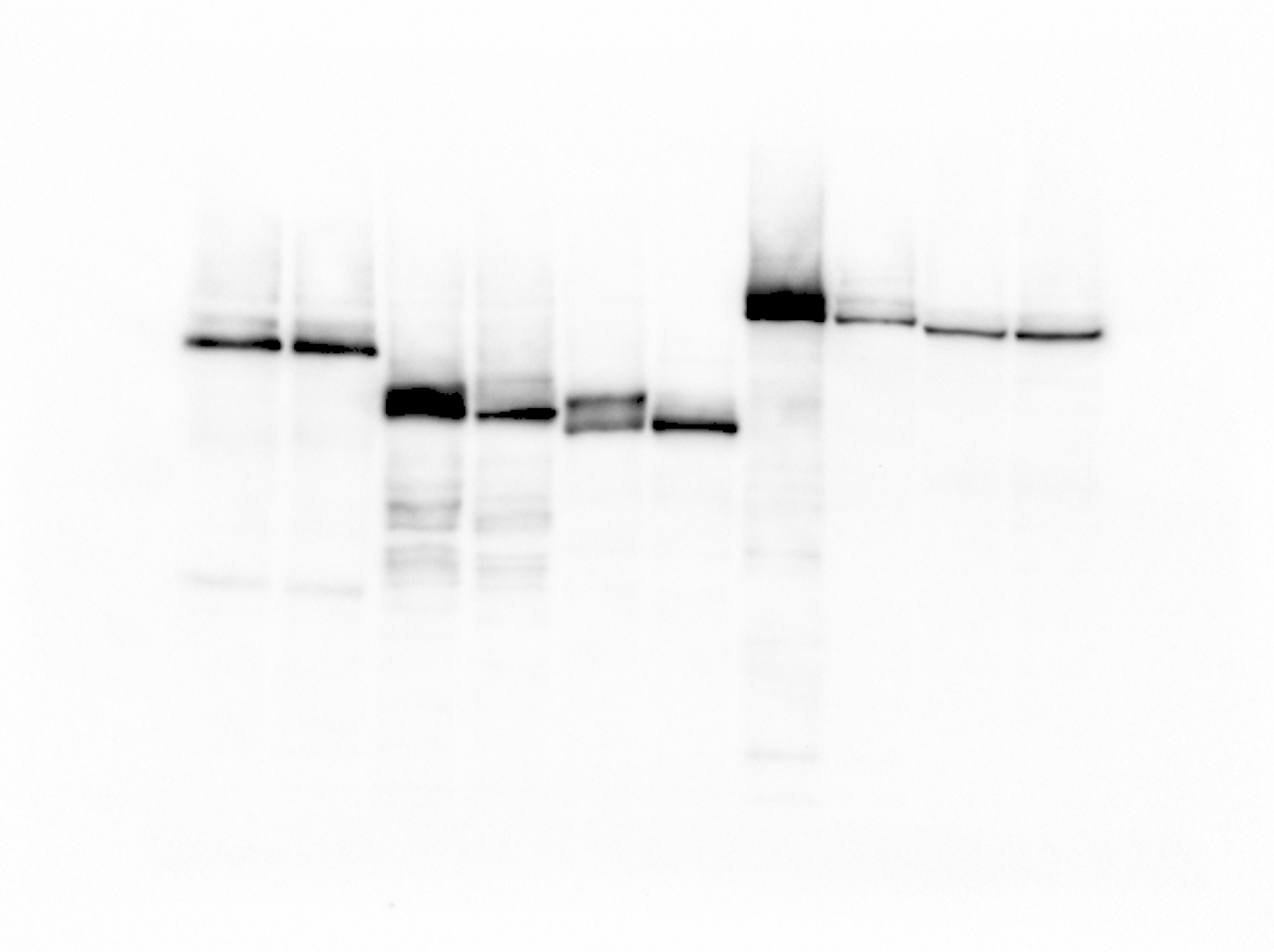

Supplement: Figure 1—figure supplement 3—source data 1. [file elife-102301-fig1-figsupp3-data1.zip › Figure 1-figure supplement 3B-V5.jpg]

### WT HEK293T cells

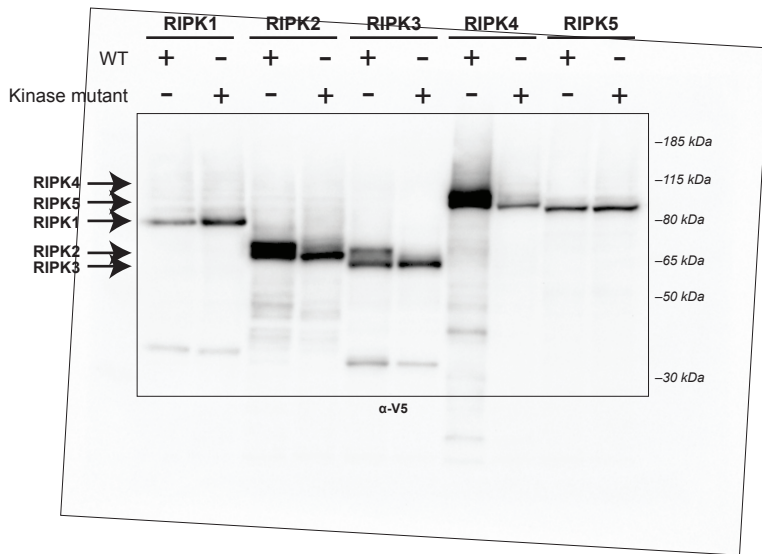

### RIPK1 KO HEK293T cells

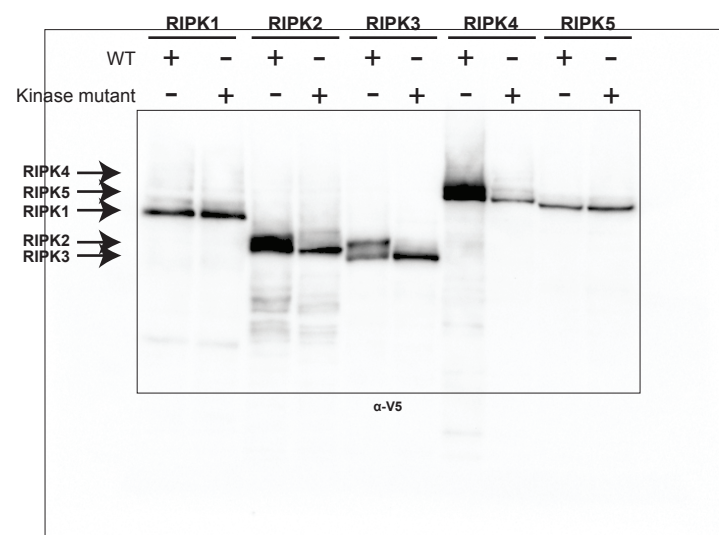

### WT HEK293T cells

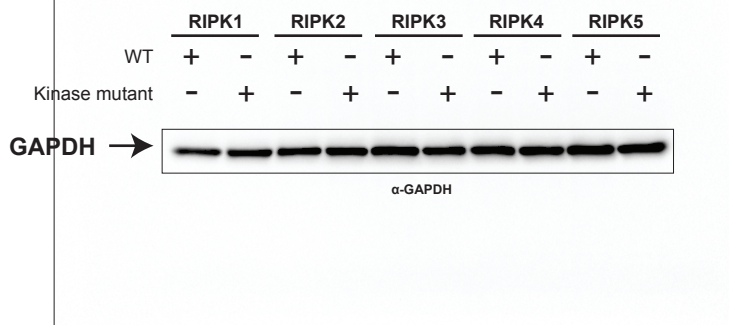

### RIPK1 KO HEK293T cells

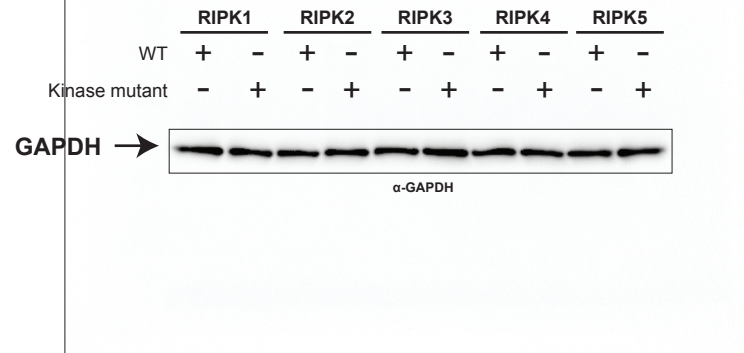

Supplement: Figure 1—figure supplement 3—source data 2. [file elife-102301-fig1-figsupp3-data2.zip › Figure 1-figure supplement 3-source-data2.pdf]

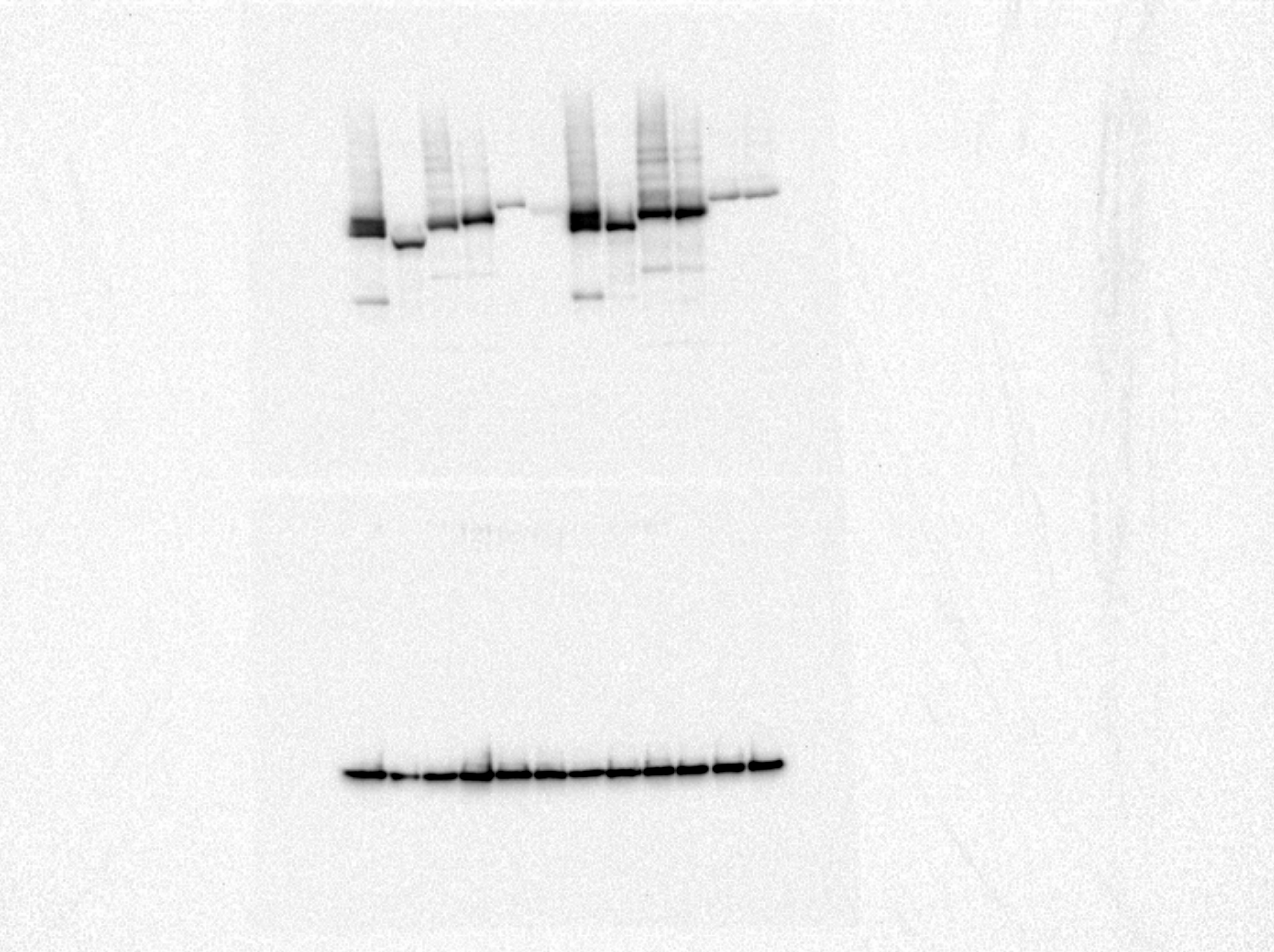

Supplement: Figure 2—figure supplement 1—source data 1. [file elife-102301-fig2-figsupp1-data1.zip › Figure 2-figure supplement 1-non-mammal RIPK3-V5 top-GAPDH bottom.jpg]

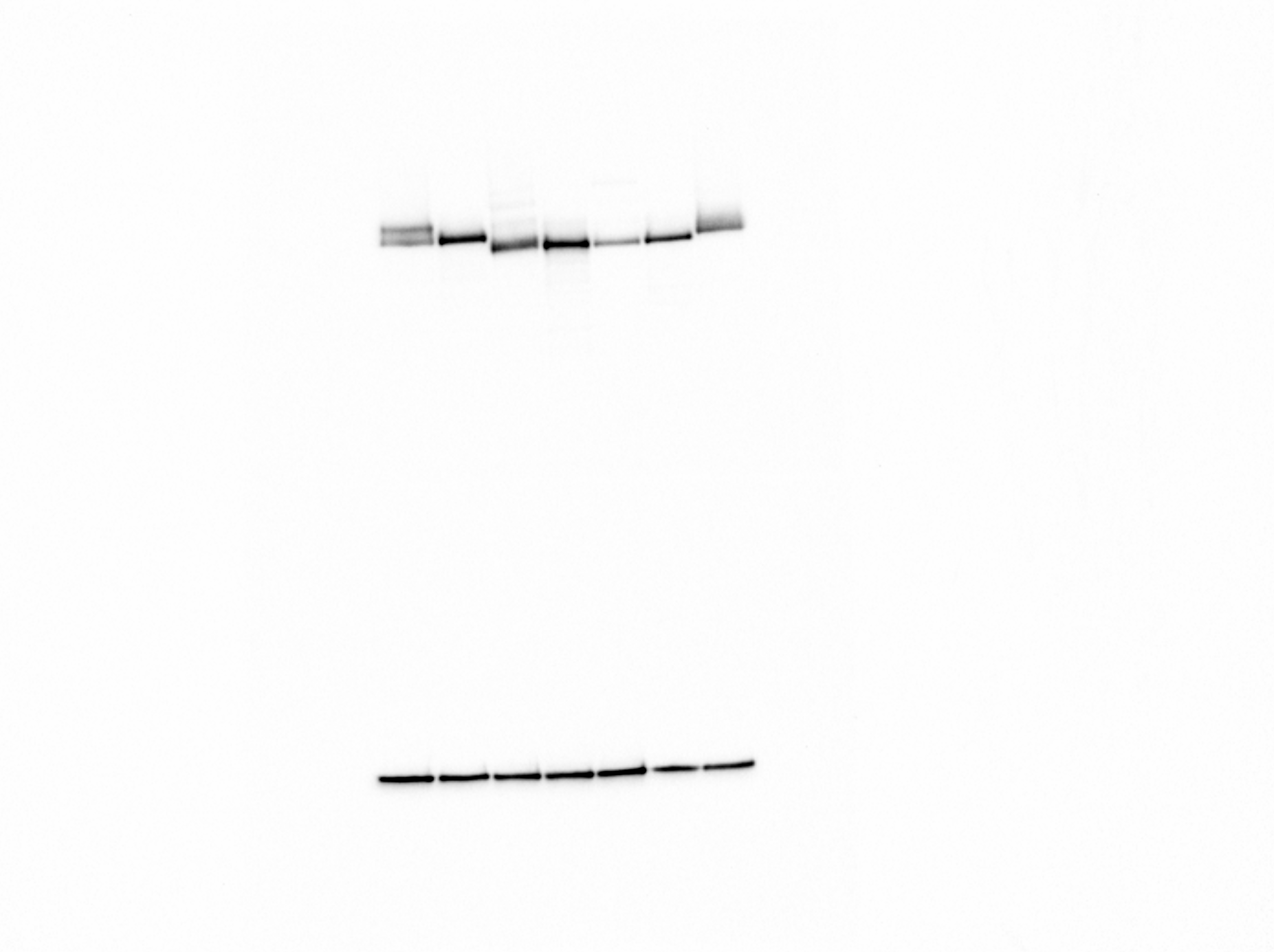

Supplement: Figure 2—figure supplement 1—source data 1. [file elife-102301-fig2-figsupp1-data1.zip › Figure 2-figure supplement 1-RIPK1 KO 293T-mammal RIPK3-V5 top-GAPDH bottom.jpg]

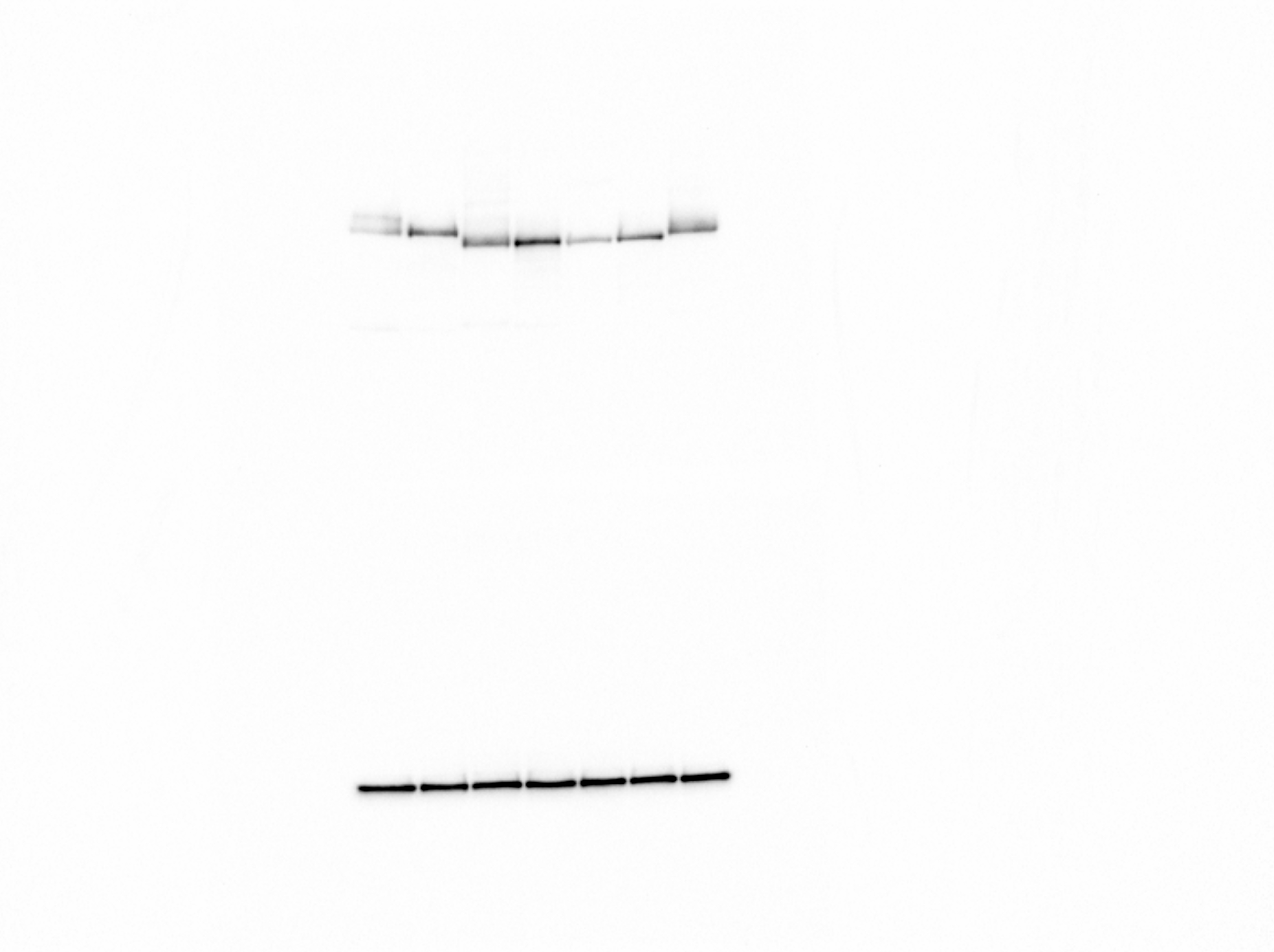

Supplement: Figure 2—figure supplement 1—source data 1. [file elife-102301-fig2-figsupp1-data1.zip › Figure 2-figure supplement 1-WT 293T-mammal RIPK3-GAPDH bottom.jpg]

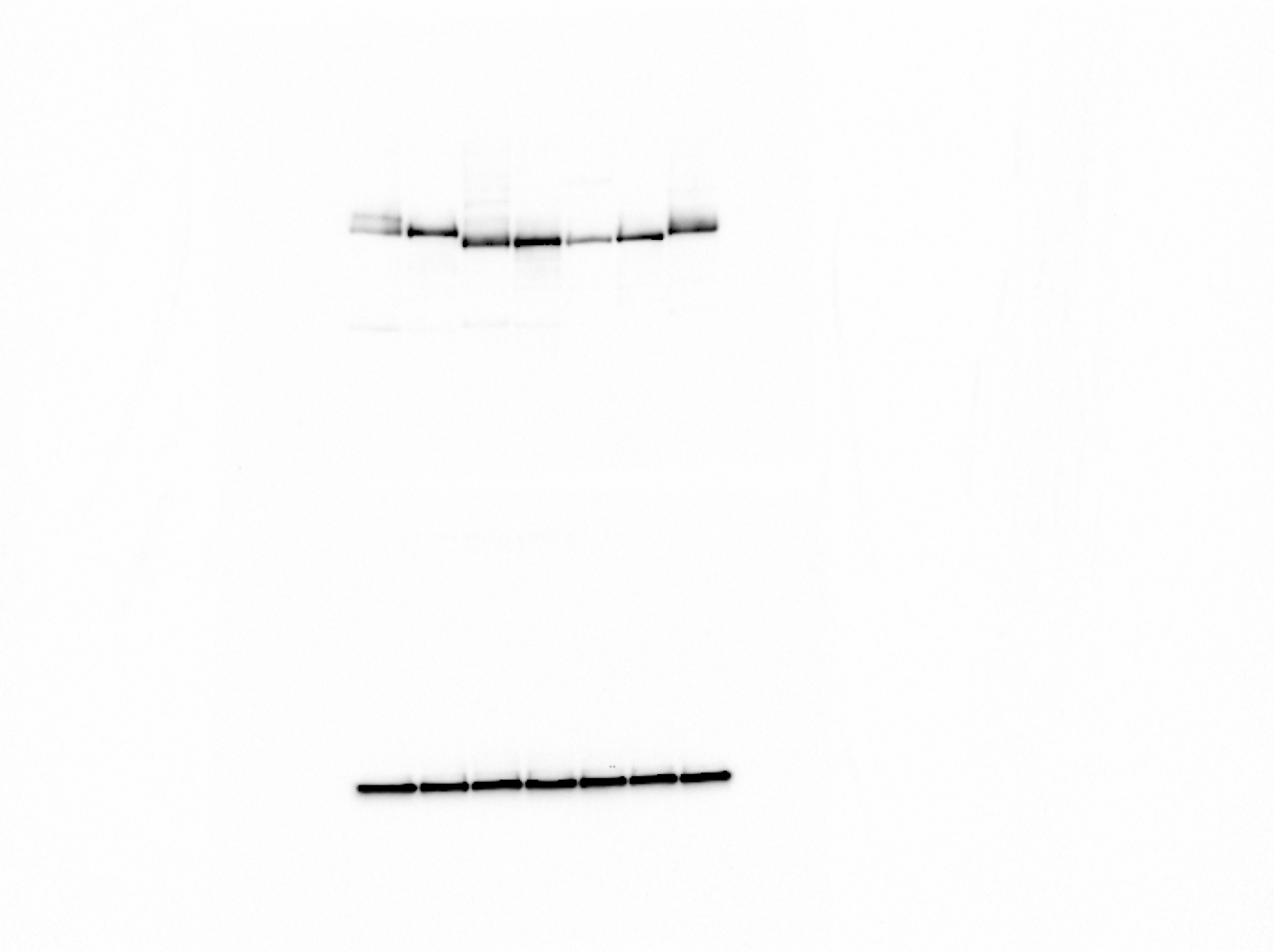

Supplement: Figure 2—figure supplement 1—source data 1. [file elife-102301-fig2-figsupp1-data1.zip › Figure 2-figure supplement 1-WT 293T-mammal RIPK3-V5 top.jpg]

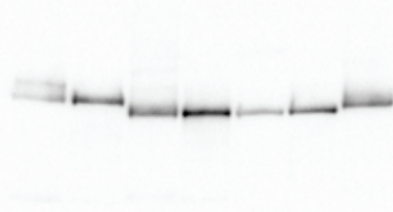

WT HEK293T:

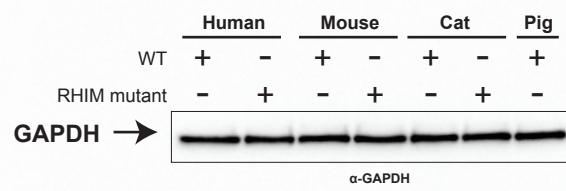

Supplement: Figure 2—figure supplement 1—source data 2. [file elife-102301-fig2-figsupp1-data2.zip › Figure 2-figure supplement 1-source-data2-1bottom.pdf]

WT HEK293T:

|             | Human |   | Mouse |   | Cat |   | Pig |
|-------------|-------|---|-------|---|-----|---|-----|
| WT          | +     | - | +     | - | +   | - | +   |
| RHIM mutant | -     | + | -     | + | -   | + | -   |

RIPK3s

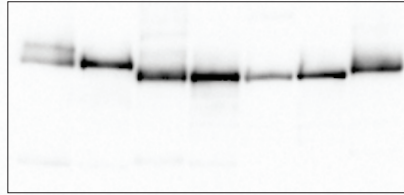

α-V5

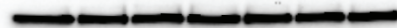

Supplement: Figure 2—figure supplement 1—source data 2. [file elife-102301-fig2-figsupp1-data2.zip › Figure 2-figure supplement 1-source-data2-1top.pdf]

**RIPK1 KO HEK293T:**

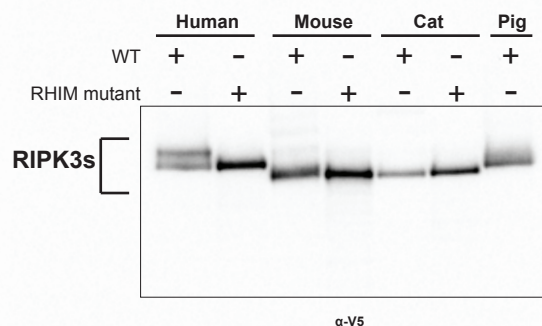

**RIPK1 KO HEK293T:**

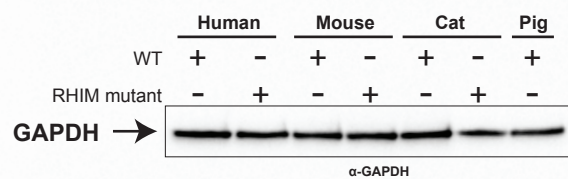

Supplement: Figure 2—figure supplement 1—source data 2. [file elife-102301-fig2-figsupp1-data2.zip › Figure 2-figure supplement 1-source-data2-2.pdf]

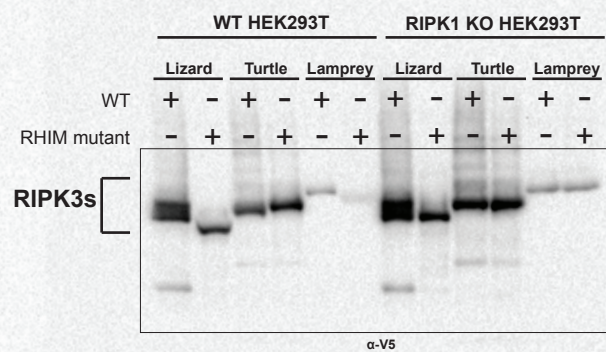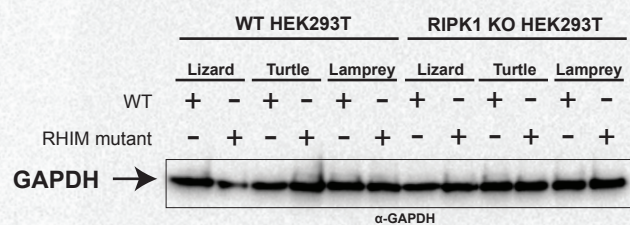

Supplement: Figure 2—figure supplement 1—source data 2. [file elife-102301-fig2-figsupp1-data2.zip › Figure 2-figure supplement 1-source-data2-3.pdf]

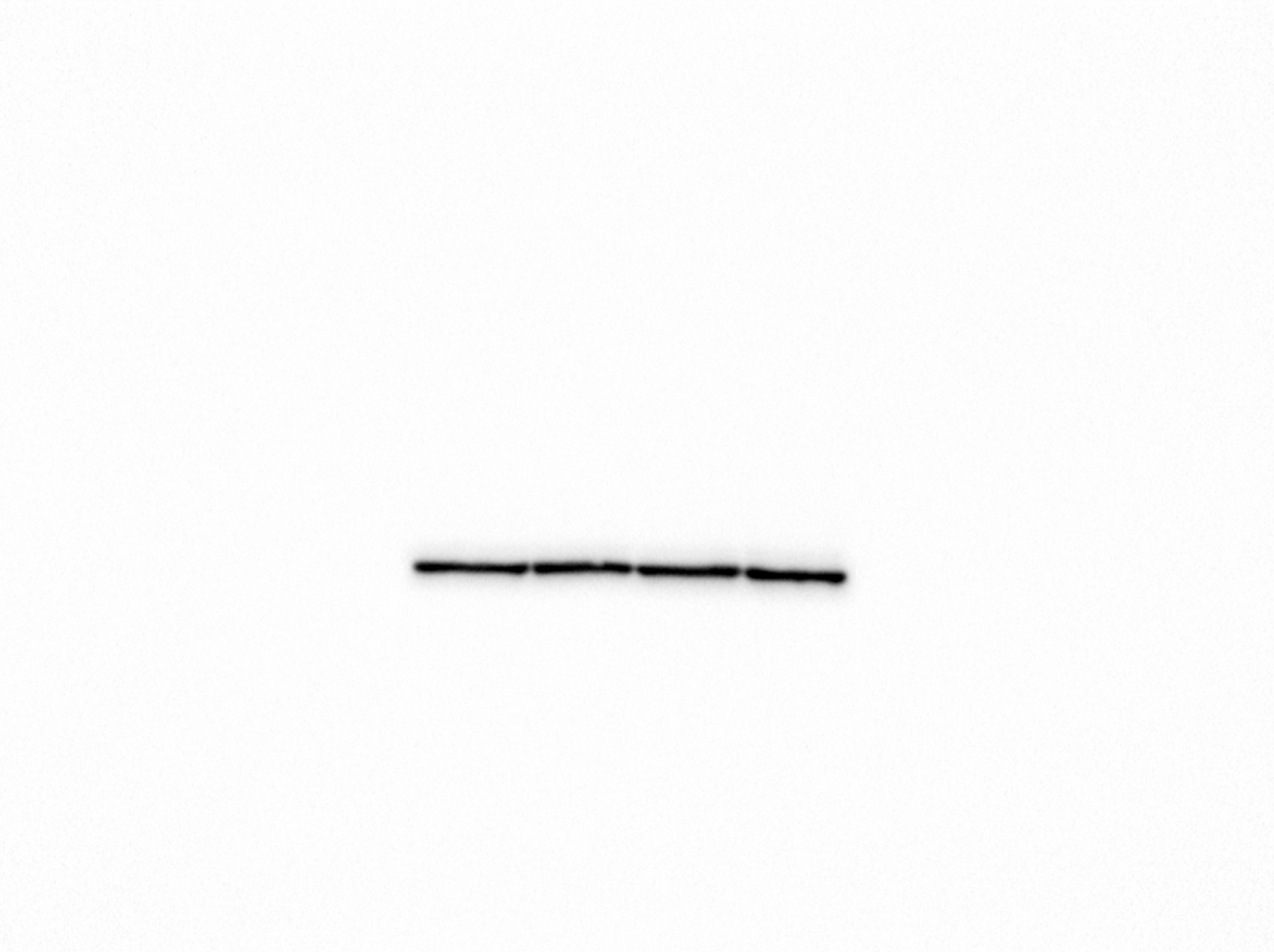

Supplement: Figure 3—figure supplement 2—source data 1. [file elife-102301-fig3-figsupp2-data1.zip › Figure 3-figure supplement 2-GAPDH.jpg]

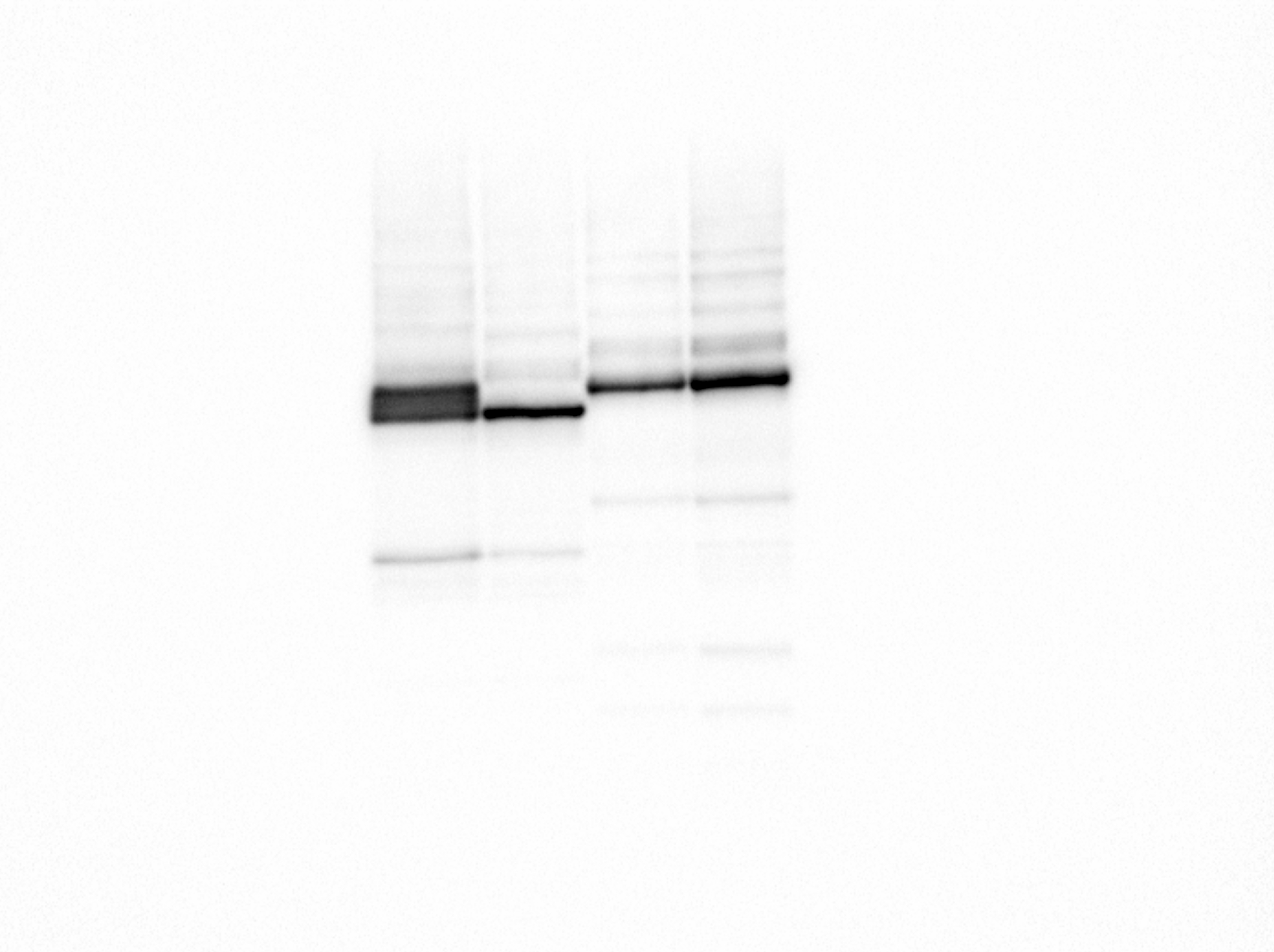

Supplement: Figure 3—figure supplement 2—source data 1. [file elife-102301-fig3-figsupp2-data1.zip › Figure 3-figure supplement 2-V5.jpg]

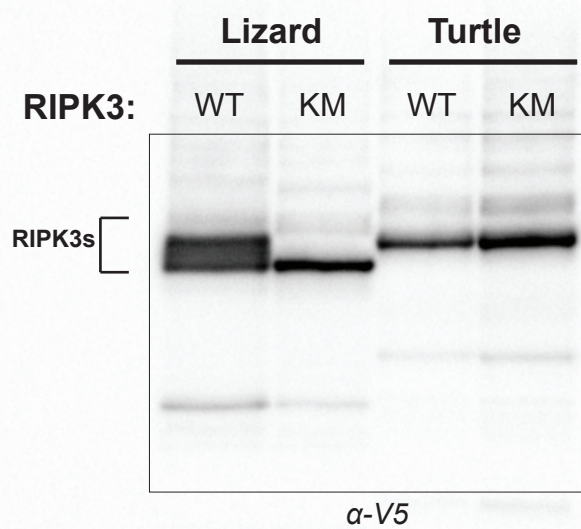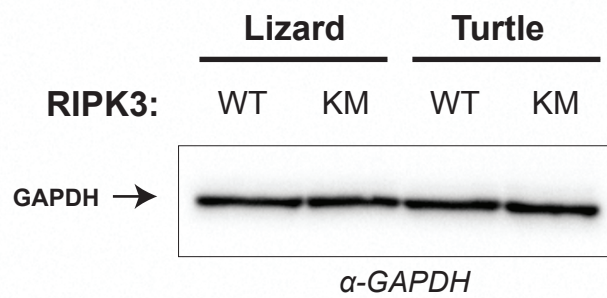

Supplement: Figure 3—figure supplement 2—source data 2. [file elife-102301-fig3-figsupp2-data2.zip › Figure 3-figure supplement 2-source-data-2.pdf]

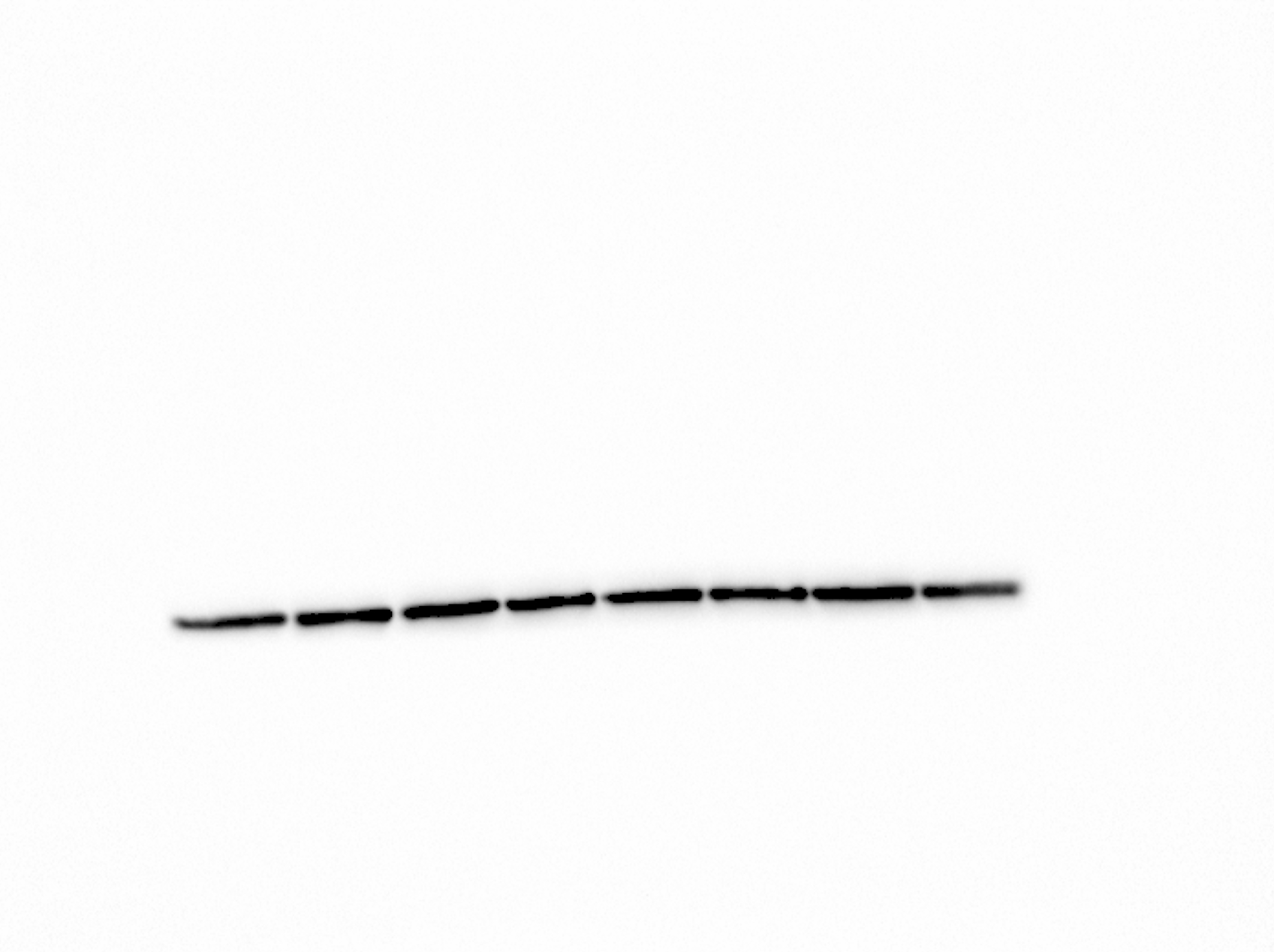

Supplement: Figure 4—figure supplement 1—source data 1. [file elife-102301-fig4-figsupp1-data1.zip › Figure 4-figure supplement 1-RIPK1 KO 293T-mammal RIPK1-GAPDH.jpg]

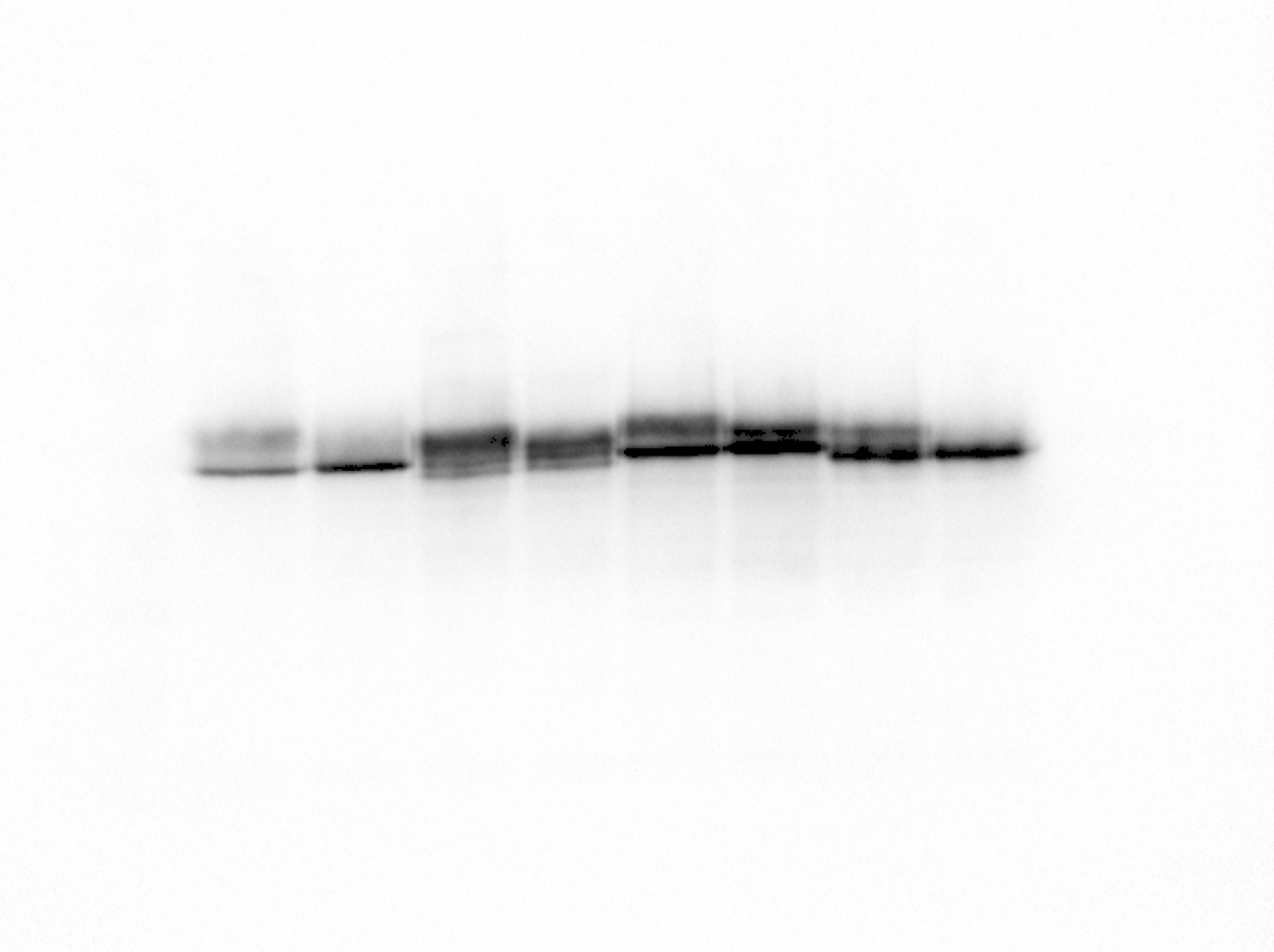

Supplement: Figure 4—figure supplement 1—source data 1. [file elife-102301-fig4-figsupp1-data1.zip › Figure 4-figure supplement 1-RIPK1 KO 293T-mammal RIPK1-V5.jpg]

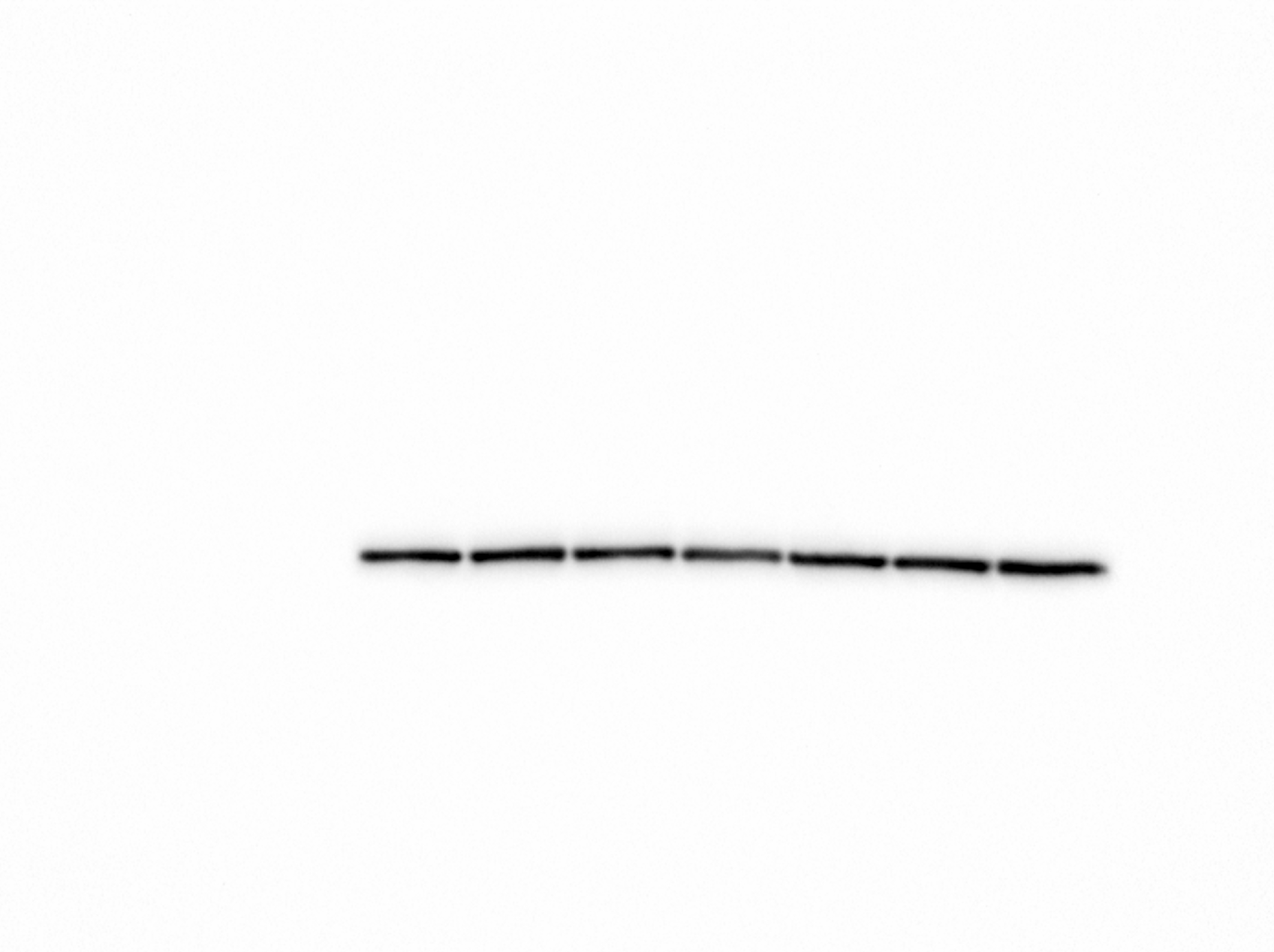

Supplement: Figure 4—figure supplement 1—source data 1. [file elife-102301-fig4-figsupp1-data1.zip › Figure 4-figure supplement 1-RIPK1 KO 293T-non-mammal RIPK1-GAPDH.jpg]

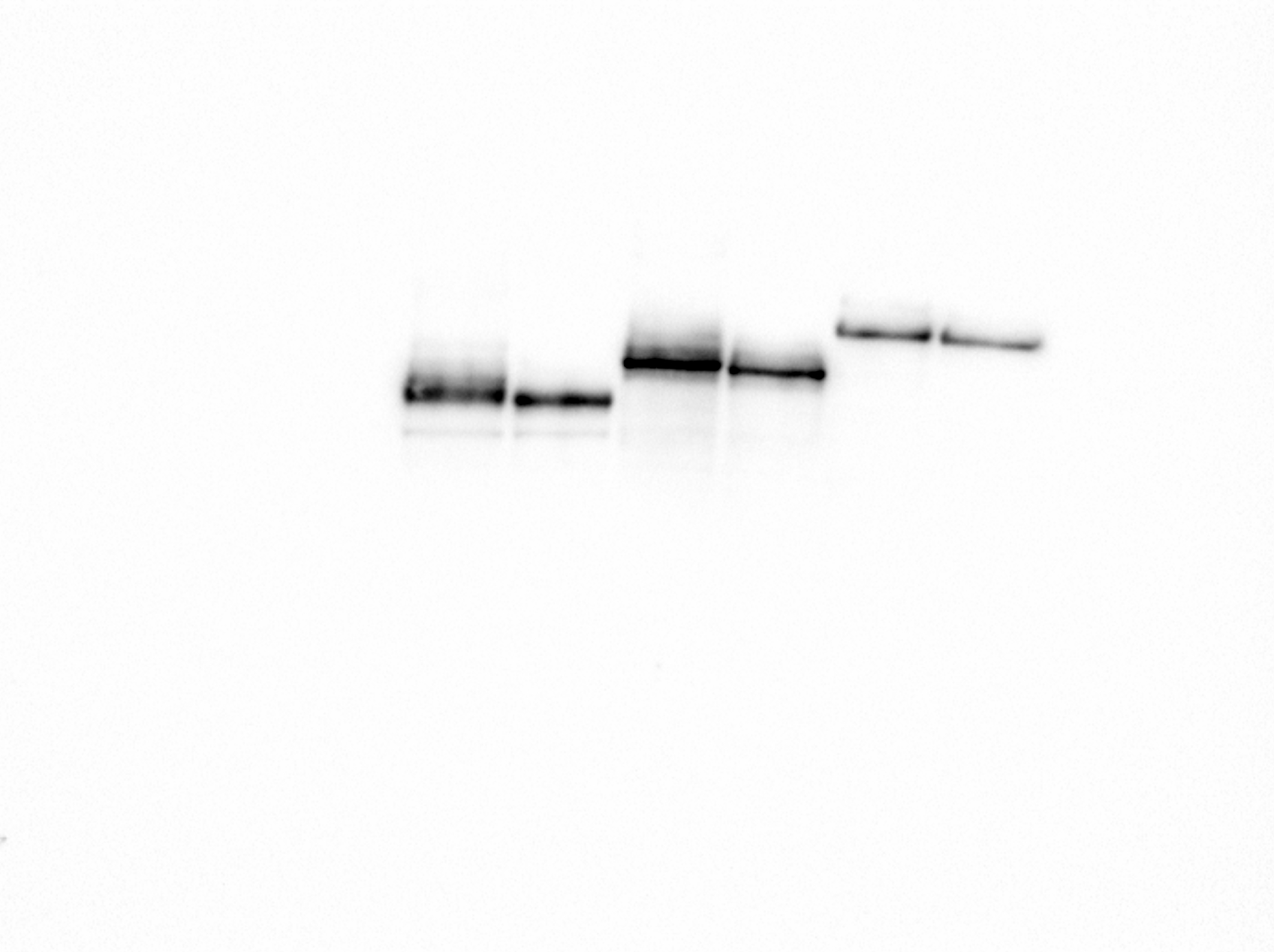

Supplement: Figure 4—figure supplement 1—source data 1. [file elife-102301-fig4-figsupp1-data1.zip › Figure 4-figure supplement 1-RIPK1 KO 293T-non-mammal RIPK1-V5.jpg]

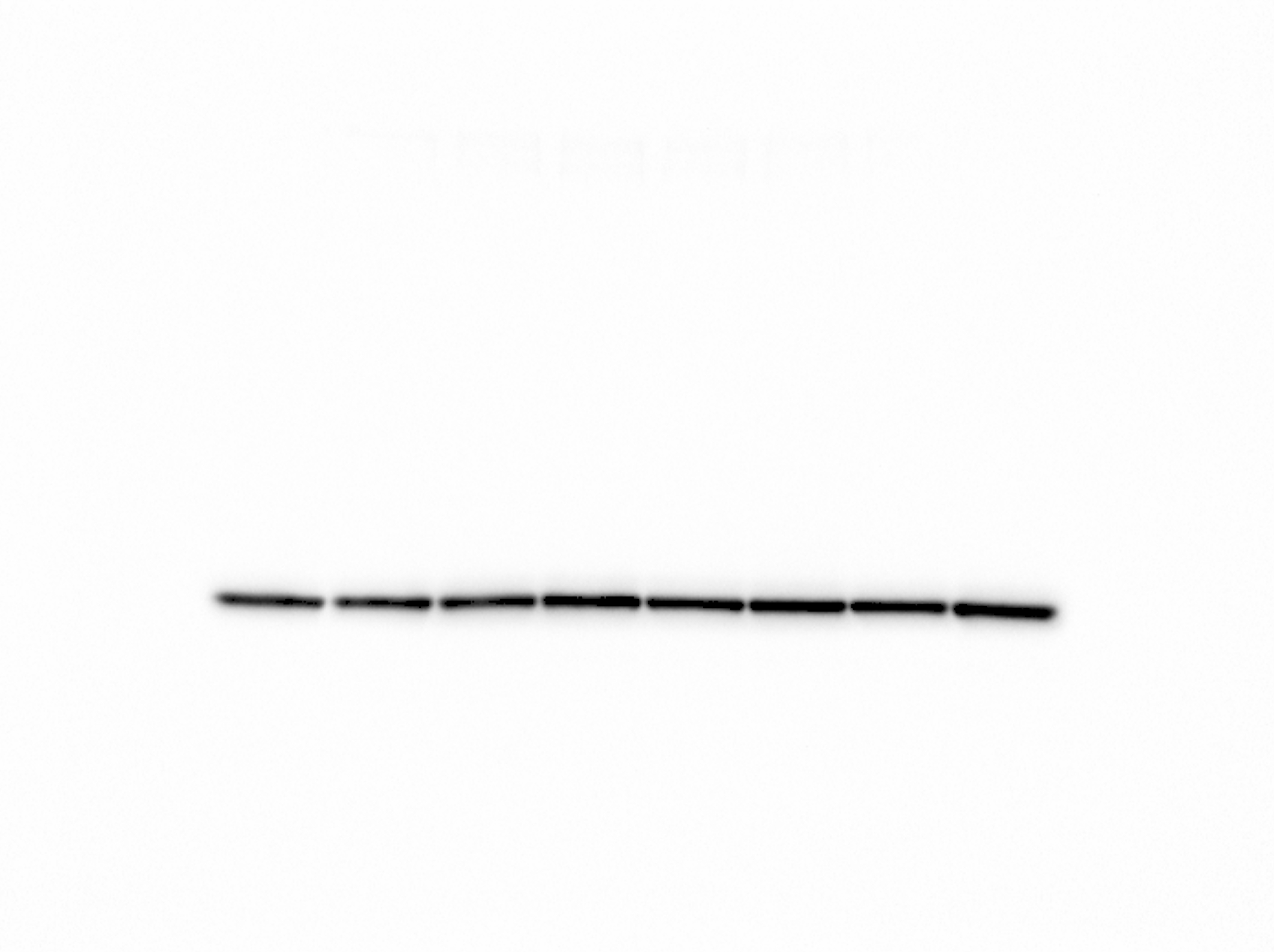

Supplement: Figure 4—figure supplement 1—source data 1. [file elife-102301-fig4-figsupp1-data1.zip › Figure 4-figure supplement 1-WT 293T-mammal RIPK1-GAPDH.jpg]

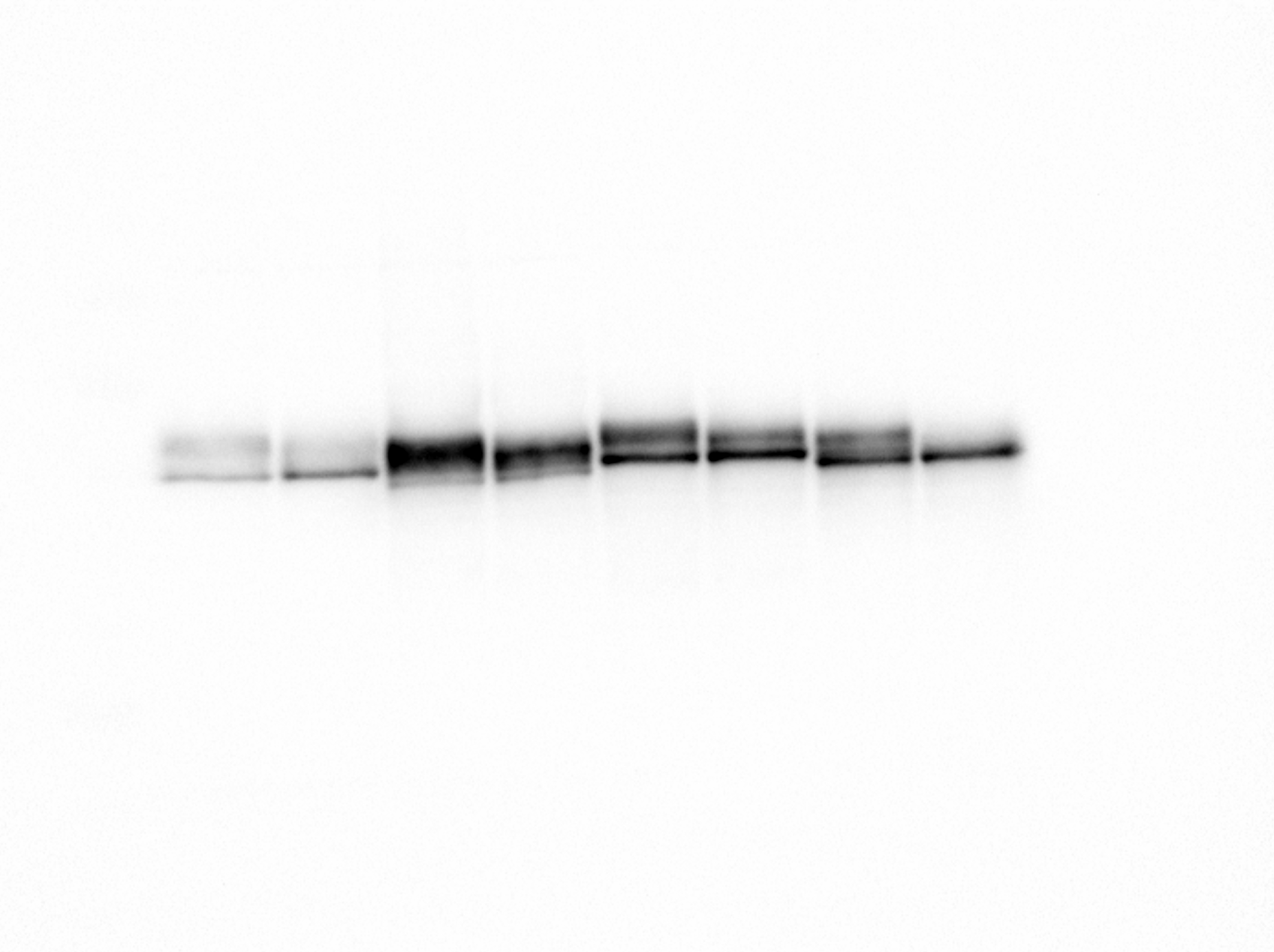

Supplement: Figure 4—figure supplement 1—source data 1. [file elife-102301-fig4-figsupp1-data1.zip › Figure 4-figure supplement 1-WT 293T-mammal RIPK1-V5.jpg]

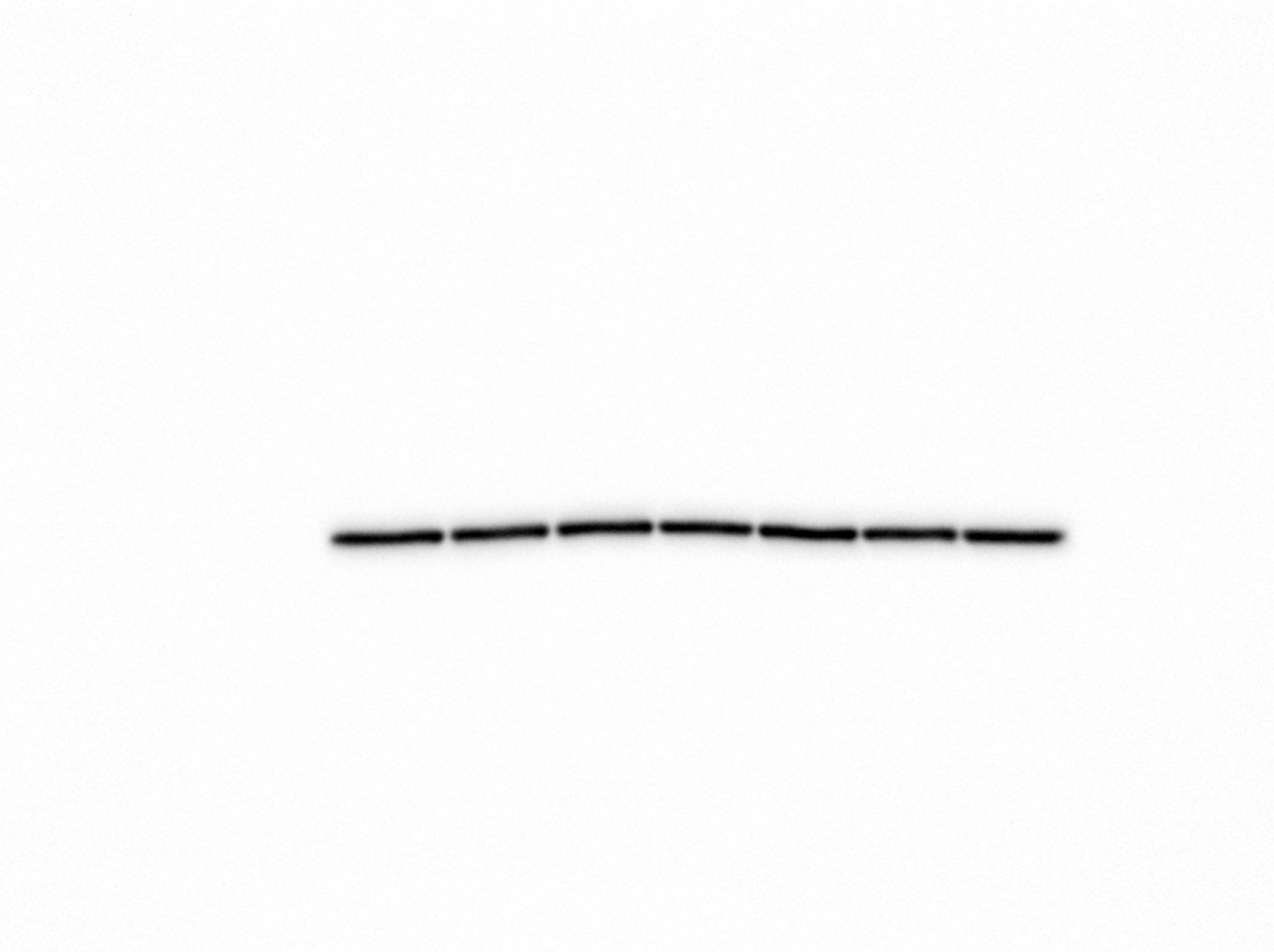

Supplement: Figure 4—figure supplement 1—source data 1. [file elife-102301-fig4-figsupp1-data1.zip › Figure 4-figure supplement 1-WT 293T-non-mammal RIPK1-GAPDH.jpg]

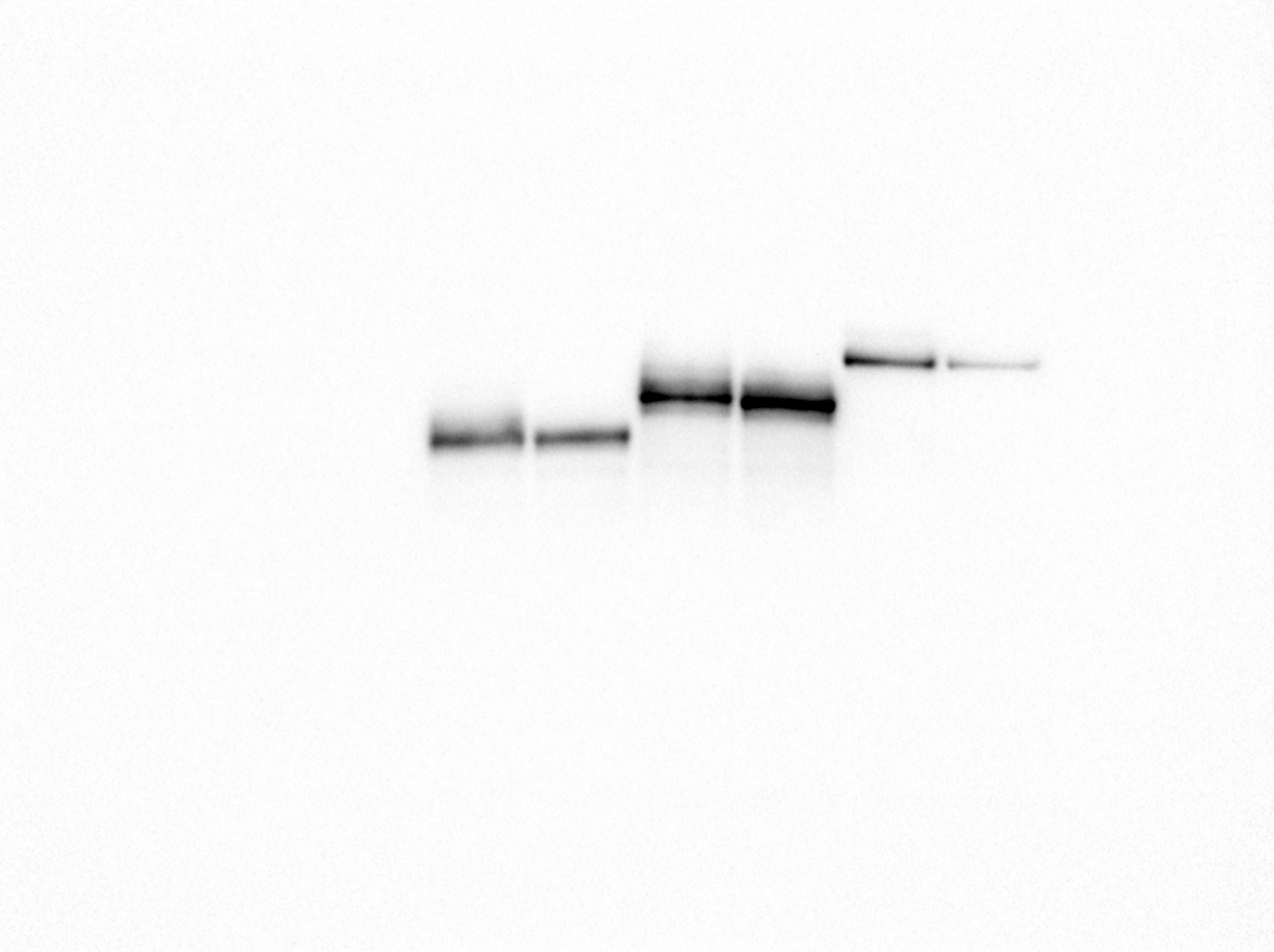

Supplement: Figure 4—figure supplement 1—source data 1. [file elife-102301-fig4-figsupp1-data1.zip › Figure 4-figure supplement 1-WT 293T-non-mammal RIPK1-V5.jpg]

WT HEK293T:

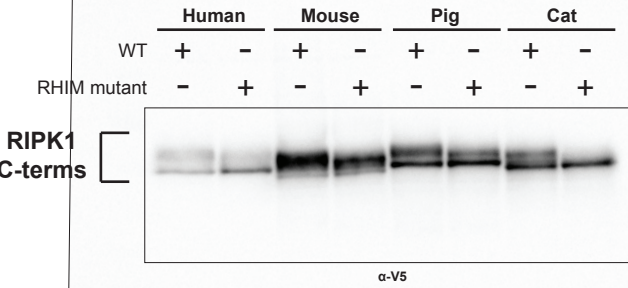

WT HEK293T:

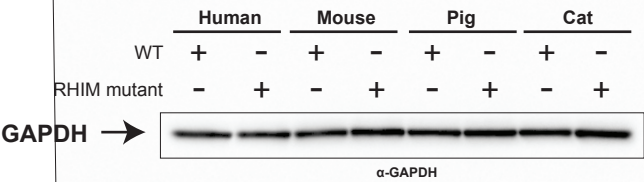

Supplement: Figure 4—figure supplement 1—source data 2. [file elife-102301-fig4-figsupp1-data2.zip › Figure 4-figure supplement 1-source-data-2-1.pdf]

**RIPK1 KO HEK293T:**

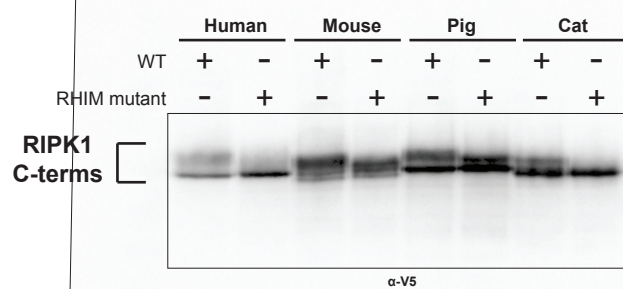

**RIPK1 KO HEK293T:**

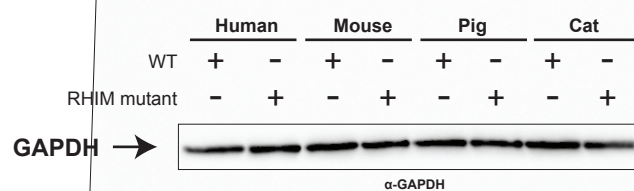

Supplement: Figure 4—figure supplement 1—source data 2. [file elife-102301-fig4-figsupp1-data2.zip › Figure 4-figure supplement 1-source-data-2-2.pdf]

**WT HEK293T:**

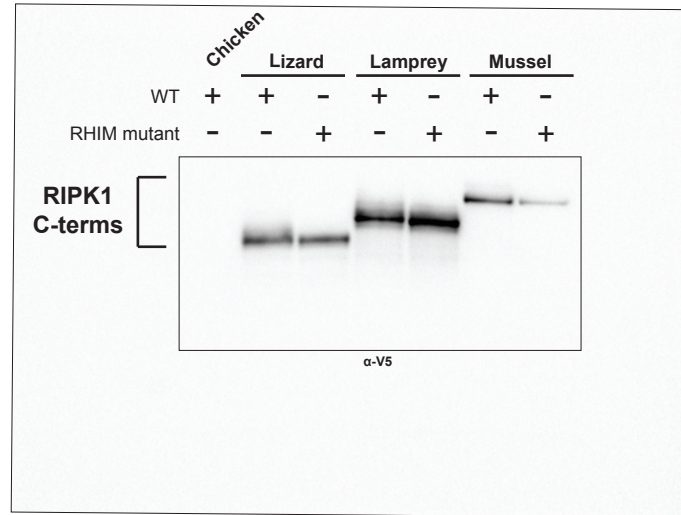

**WT HEK293T:**

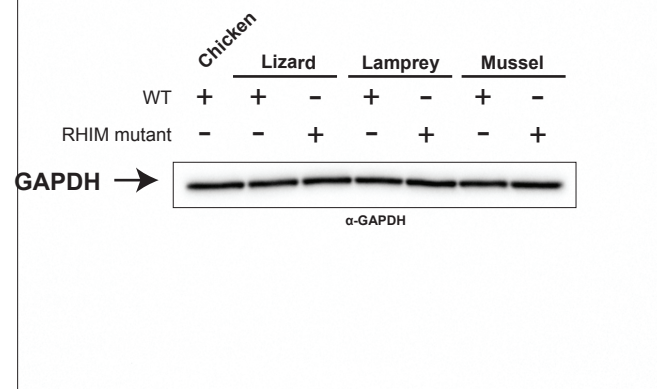

Supplement: Figure 4—figure supplement 1—source data 2. [file elife-102301-fig4-figsupp1-data2.zip › Figure 4-figure supplement 1-source-data-2-3.pdf]

**RIPK1 KO HEK293T:**

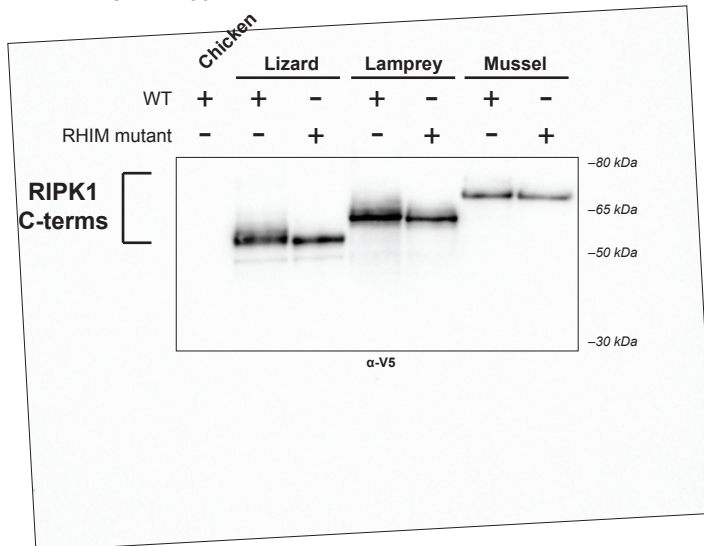

**RIPK1 KO HEK293T:**

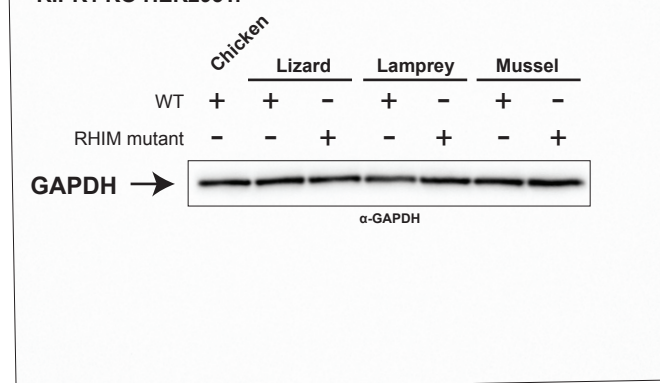

Supplement: Figure 4—figure supplement 1—source data 2. [file elife-102301-fig4-figsupp1-data2.zip › Figure 4-figure supplement 1-source-data-2-4.pdf]

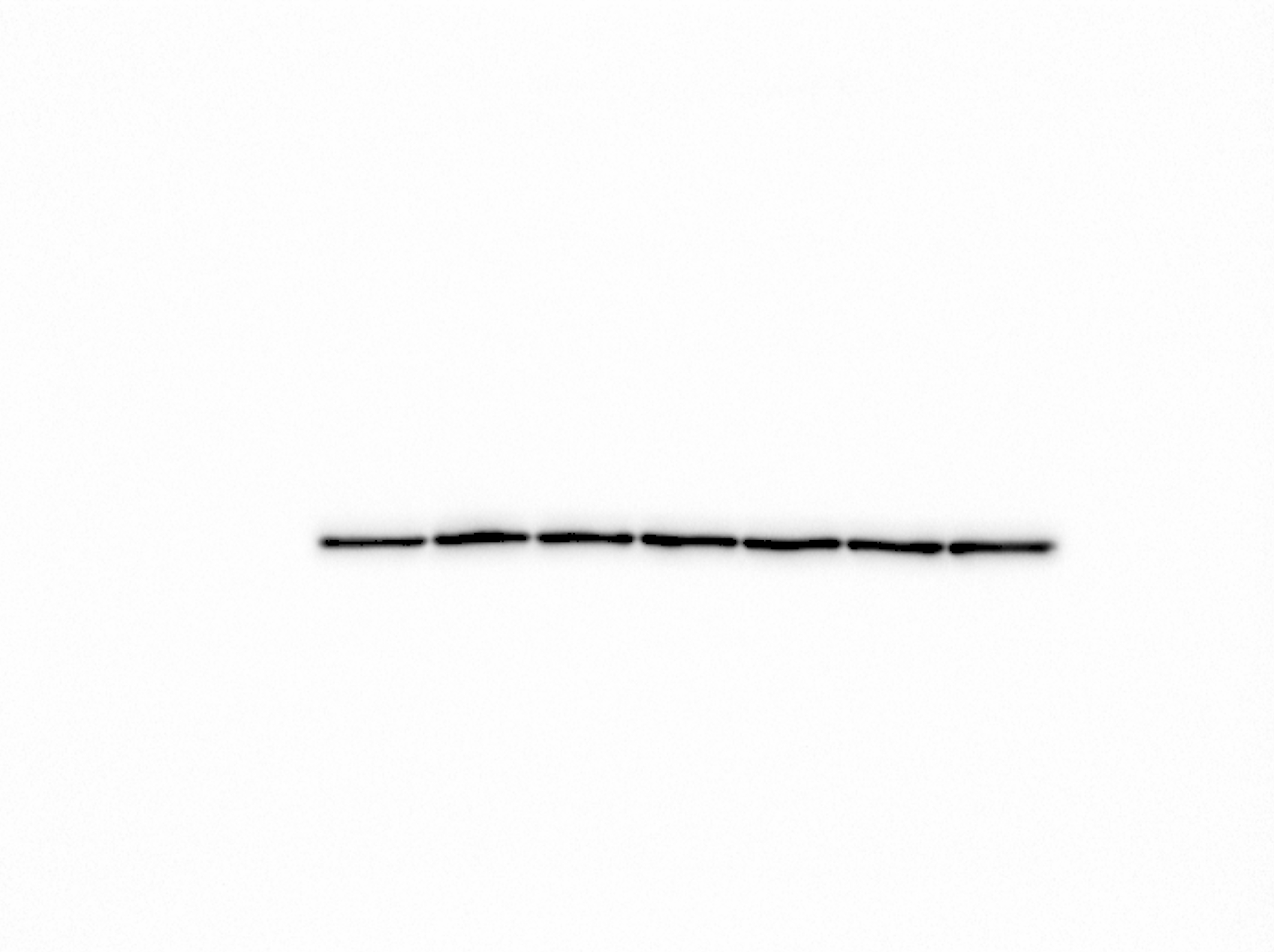

Supplement: Figure 4—figure supplement 6—source data 1. [file elife-102301-fig4-figsupp6-data1.zip › Figure 4-figure supplement 6-GAPDH.jpg]

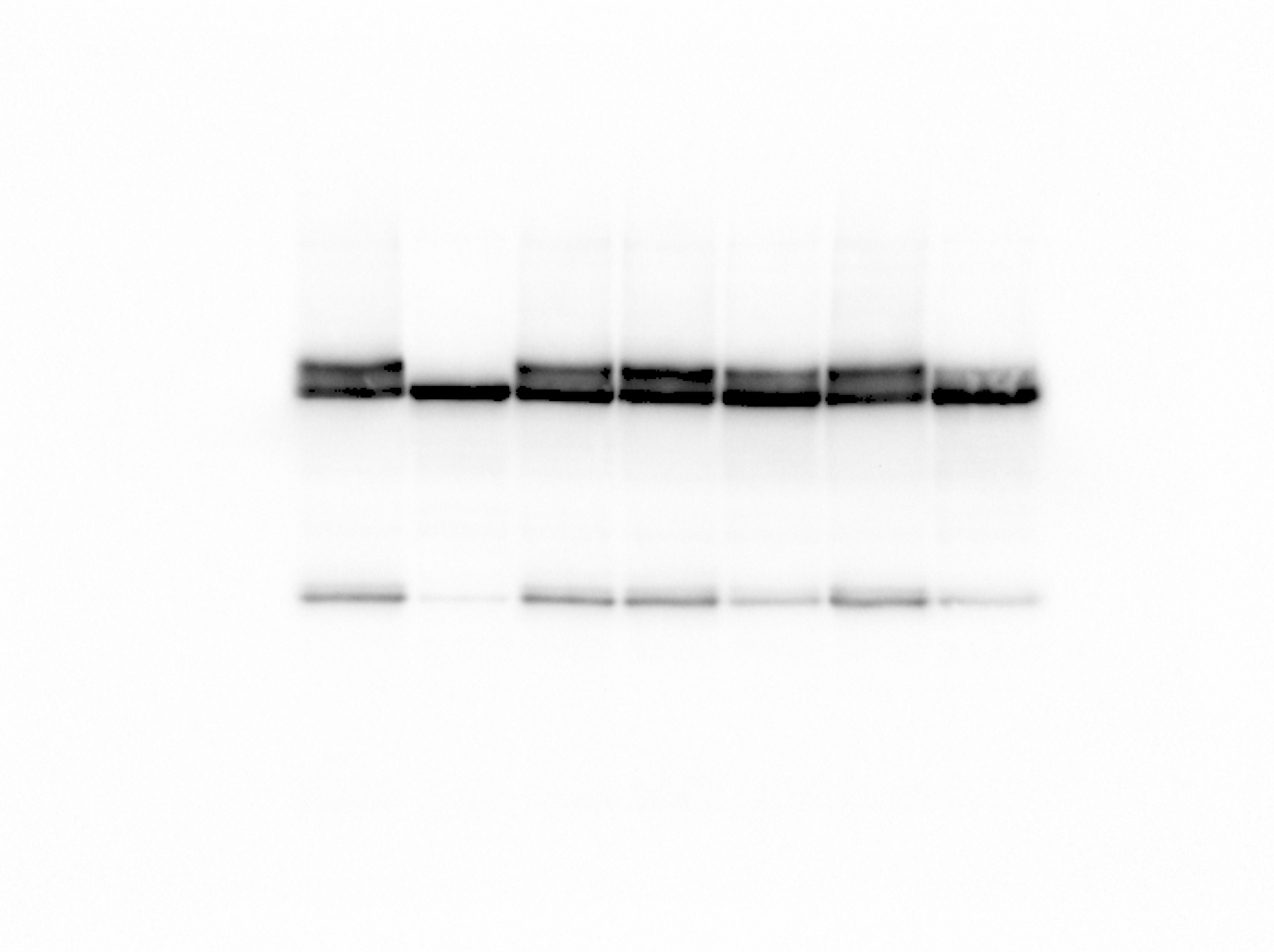

Supplement: Figure 4—figure supplement 6—source data 1. [file elife-102301-fig4-figsupp6-data1.zip › Figure 4-figure supplement 6-V5.jpg]

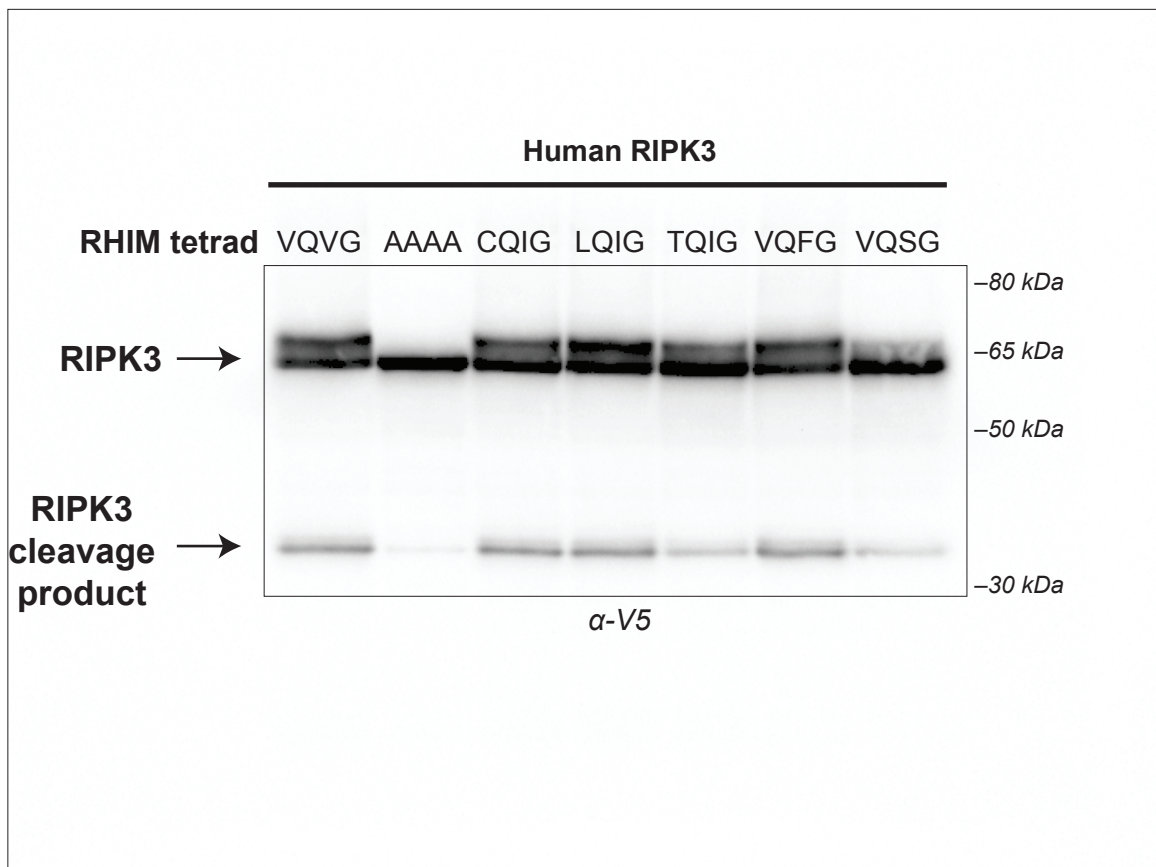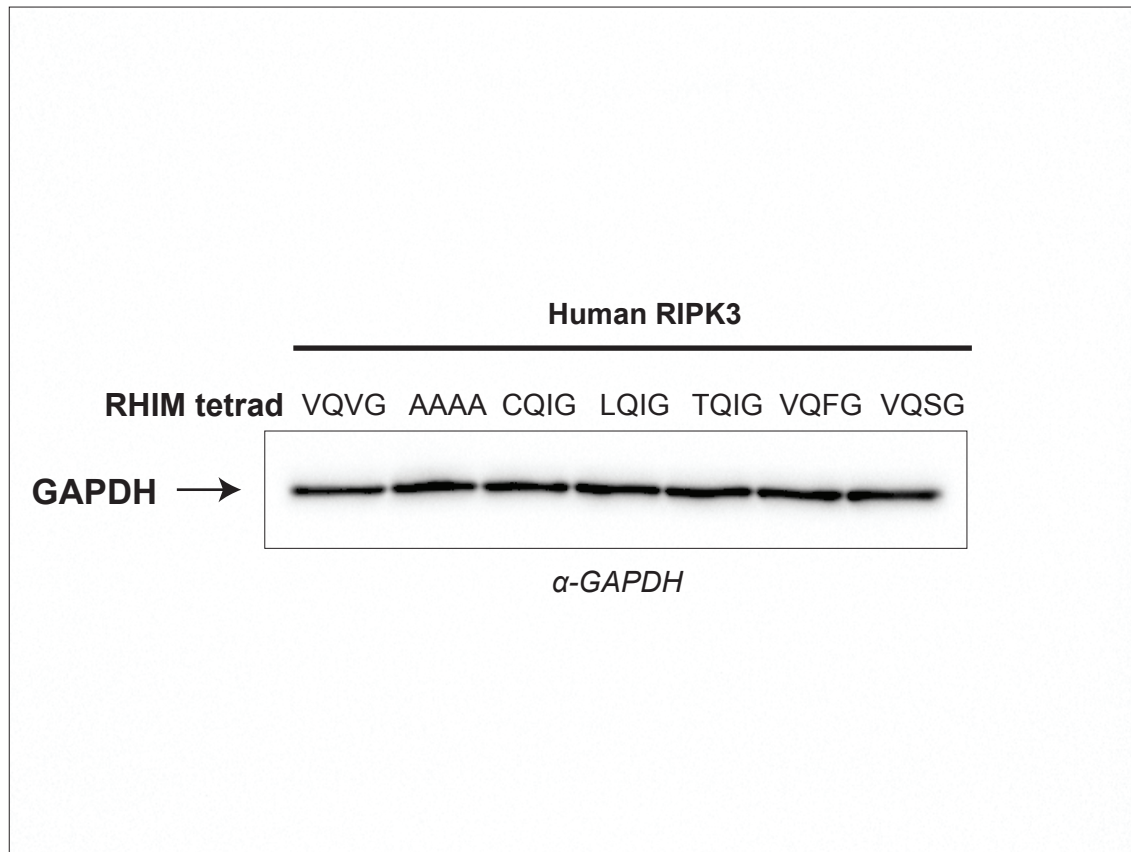

Supplement: Figure 4—figure supplement 6—source data 2. [file elife-102301-fig4-figsupp6-data2.zip › Figure 4-figure supplement 6-source-data-2.pdf]
